# Controlled Magnesium Ion Delivery via Mg‐Sputtered Nerve Conduit for Enhancing Peripheral Nerve Regeneration

**DOI:** 10.1002/adhm.202500063

**Published:** 2025-04-27

**Authors:** Hyewon Kim, Jieun Kwon, Hyeok Kim, Sunhee Lee, Seongchan Kim, Ji‐Young Lee, Khandoker Asiqur Rahaman, Taeyeon Kim, Hyojin Lee, Myoung‐Ryul Ok, Seok Chung, Hyung‐Seop Han, Yu‐Chan Kim

**Affiliations:** ^1^ Biomaterials Research Center Biomedical Research Division Korea Institute of Science and Technology (KIST) Seoul 02792 Republic of Korea; ^2^ Department of Biomicro System Technology Korea University Seoul 02841 Republic of Korea; ^3^ College of Pharmacy and Research Institute of Pharmaceutical Sciences Gyeongsang National University Jinju Gyeongsangnam‐do 52828 Republic of Korea; ^4^ Division of Bio‐Medical Science & Technology KIST School University of Science and Technology (UST) Seoul 02792 Republic of Korea; ^5^ SKKU‐KIST Department of Integrative Biotechnology College of Biotechnology and Bioengineering Sungkyunkwan University Gyeonggi Suwon 16419 South Korea

**Keywords:** controlled degradation, magnesium, nerve conduits, peripheral nerve regeneration

## Abstract

Autologous nerve grafting remains the gold standard for treating peripheral nerve injuries; however, it is constrained by limited donor nerve availability, the need for secondary surgeries, and sensory loss at the donor site. Biodegradable material‐based nerve conduits have emerged as a promising alternative to address these limitations and enhance nerve regeneration. Among these materials, magnesium stands out due to its exceptional biocompatibility, biofunctionality, and neuroprotective properties. Despite its potential, magnesium's rapid corrosion rate and the need for controlled ion release necessitate advanced modifications, such as the development of Mg alloys. However, these approaches often face challenges, including viability concerns and material hardness, which can hinder nerve repair and damage surrounding tissues. In this study, a novel solution is introduced by sputtering magnesium onto a soft collagen sheet, achieving controlled magnesium ion release while preserving the material's nerve‐like softness. This Mg‐sputtered collagen sheet demonstrates excellent biocompatibility and significantly improves axon regeneration, muscle reinnervation, and functional recovery in a sciatic nerve defect model. These findings highlight the potential of an innovative Mg‐based biodegradable nerve conduit, offering transformative applications across various medical fields.

## Graphical abstract

This study introduces a controllable degradation system for Mg‐based biomaterials using sputtering technology, marking a significant advancement in nerve regeneration research. The Mg‐sputtered nerve conduits demonstrate enhanced biocompatibility, biofunctionality, mechanical compatibility, and precise magnesium release, resulting in improved axonal regeneration and functional recovery. These findings underscore the potential of CPL@3Mg conduits as a superior alternative to traditional autografts, offering an innovative and effective solution for peripheral nerve repair.

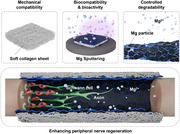

## 1 Introduction

Peripheral nerve injuries resulting from trauma, diabetes, or accidents pose a significant clinical challenge, often leading to a substantial decline in a patient's quality of life.^[^ ^1^ ^]^ Although autologous nerve grafting remains the gold standard for treating such injuries, it has several drawbacks, including the limited availability of donor nerves, the necessity for secondary surgical procedures, sensory deficits, and pain at the donor site.^[^ ^2^ ^]^ Consequently, there is an urgent need for alternative therapeutic strategies that can overcome these limitations and improve clinical outcomes.^[^ ^3^ ^]^

In recent years, biofunctional ions have garnered considerable attention as a promising agent for bridging nerve defects and facilitating repair due to their bioactivity and biochemical signaling properties.^[^ ^4^ ^]^ Ions, such as Mg^2+^,^[^ ^5^ ^]^ Zn^2+^,^[^ ^6^ ^]^ Li^2+^,^[^ ^7^ ^]^ and Ca^2+[^ ^8^ ^]^ have been shown to promote neurogenesis directly or stimulate other essential biological processes, including vascularization, immunomodulation, and neuroprotection, thereby supporting peripheral nerve regeneration.^[^ ^9^ ^]^ Magnesium (Mg), an essential mineral and biofunctional ion, plays a vital role in various biological processes, including deoxyribonucleic acid and ribonucleic acid synthesis and protein metabolism.^[^ ^10^ ^]^ It has been widely applied in medical devices, such as implants and stents, due to its exceptional mechanical properties.^[^ ^11^, ^12^ ^]^ Furthermore, Mg effectively mitigates excitotoxicity, inflammation, and oxidative stress, reducing nerve damage and demonstrating considerable potential for peripheral nerve regeneration.^[^ ^13^, ^14^ ^]^ Mg ions (Mg^2+^) enhance cellular attachment, support short‐gap nerve growth, and facilitate nerve regeneration after crush injuries.^[^ ^15^, ^16^ ^]^ However, despite its benefits, Mg presents significant challenges in medical applications. In physiological environments, Mg degrades, releasing Mg^2+^ ions and hydrogen gas.^[^ ^17^ ^]^ Uncontrolled degradation can result in excessive hydrogen accumulation, tissue necrosis, and impaired healing.^[^ ^18^ ^]^ Moreover, elevated Mg^2+^ levels can lead to hypermagnesemia and other adverse cellular effects. Thus, controlling the degradation rate and ion concentration of Mg is critical to its successful application in medical treatments.

Thus far, various approaches, including surface coating, alloying, and composite material development, have been explored to address the challenges associated with magnesium‐based nerve conduits. For instance, attempts to control magnesium degradation have included coating AZ91D alloy coated with carbon nanotubes (CNTs)‐calcium phosphate (CaP)/chitosan (CS),^[^ ^19^ ^]^ developing Mg‐1.6Li thin films^[^ ^20^ ^]^ and creating Li–Mg–Si bioceramics^[^ ^21^ ^]^ using an electrospinning process using polycaprolactone (PCL). However, these materials often incorporate aluminum (Al) or lithium (Li), raising potential neurotoxicity concerns. Al has been linked to neurodegenerative diseases, such as Alzheimer's and Parkinson's disease,^[^ ^22^ ^]^ while Li, though widely used to treat bipolar disorder and known for its neuroprotective effects (e.g., upregulating anti‐apoptotic factors and growth factors and reducing inflammation),^[^ ^23^, ^24^ ^]^ is not naturally present in the human body, and its potentially toxic effects remain controversial.^[^ ^25^ ^]^ To mitigate these concerns, an alternative strategy involved coating pure Mg with glial cell line‐derived neurotrophic factor (GDNF)‐gelatin methacryloyl (gel)/hydroxylapatite (HA) to promote peripheral nerve repair.^[^ ^26^ ^]^ This approach enhanced corrosion resistance, biocompatibility, and nerve regeneration. However, pure Mg exhibits Young's modulus of ≈45 GPa,^[^ ^27^ ^]^ which is ≈450 000 times higher than nerve tissue (≈100 kPa).^[^ ^28^ ^]^ This pronounced mismatch in mechanical strength can lead to inflammation and tissue damage, potentially exacerbating nerve injury.^[^ ^29^ ^]^ These findings underscore the necessity of developing biocompatible materials capable of controlled Mg^2+^ release and mechanically matched properties to ensure effective peripheral nerve regeneration.

**Figure 1:**
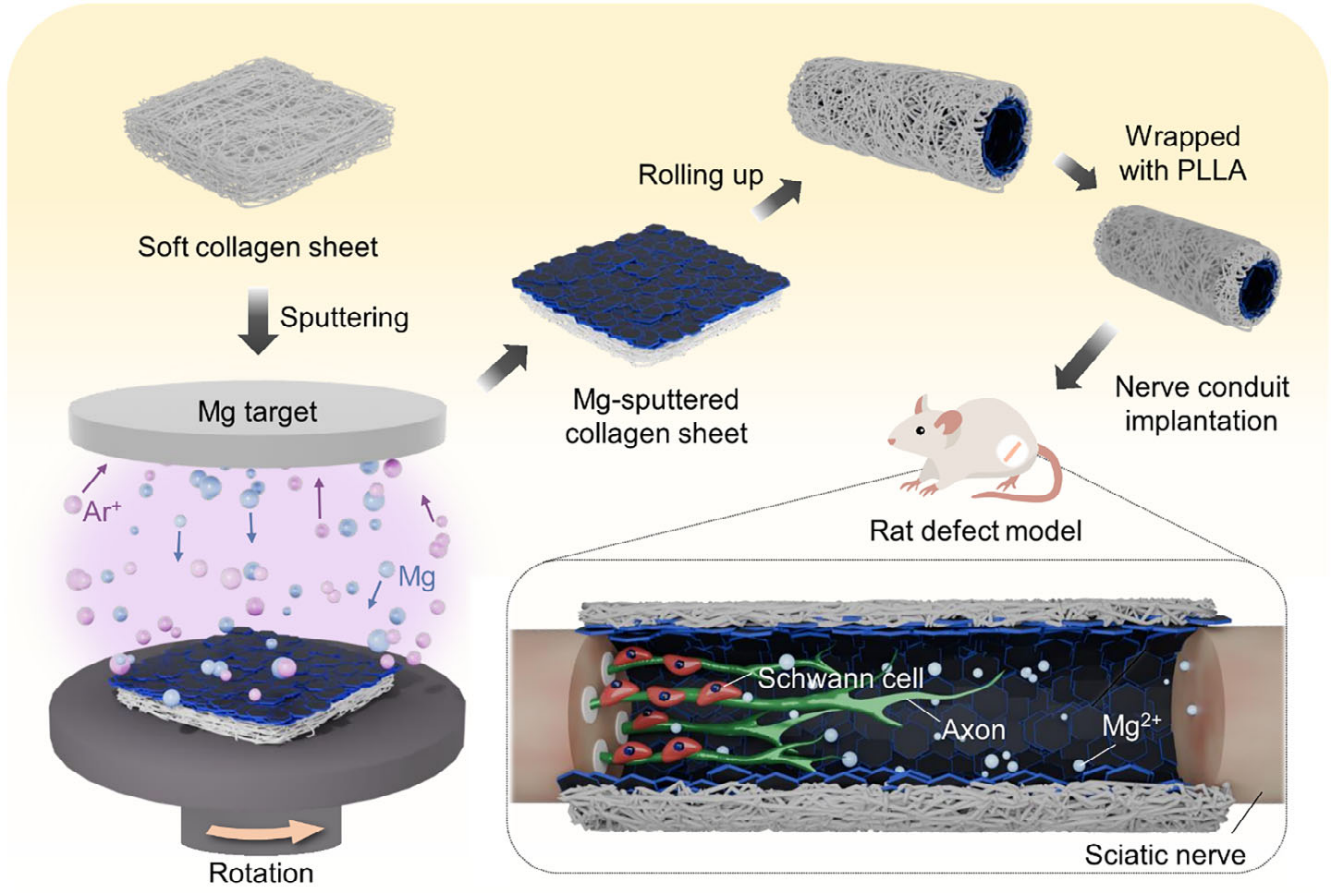
Schematic illustration of magnesium nerve conduit promoting peripheral nerve regeneration. The Mg‐sputtered nerve conduit, developed by sputtering Mg onto soft collagen sheets, enables controlled Mg ion release, supporting axonal regeneration and functional recovery in a rat defect model.

In this study, we developed an innovative Mg‐sputtered collagen nerve conduit to facilitate peripheral nerve regeneration (**Figure** **1**). Collagen, widely recognized for its biocompatibility, biodegradability, suitability for drug and ion delivery, and tunable mechanical properties, served as the base material for the nerve conduit.^[^ ^30^, ^31^ ^]^ Using sputtering, we deposited pure Mg onto the collagen scaffold, successfully incorporating bioactive ions into the conduit. The fibrous structure, mechanical properties, and degradability of the Mg‐sputtered collagen were systematically characterized. Additionally, we demonstrated its biocompatibility and potential to support neurite outgrowth in vitro. The efficacy of Mg‐sputtered collagen nerve conduit in promoting axon regeneration was evaluated in vivo by repairing nerve defects. Functional nerve healing was assessed through walking analysis, electrophysiological analysis, and histological tissue examination. Our findings introduce an innovative, bioactive nerve conduit that offers a promising solution for the repair and functional recovery of challenging peripheral nerve injuries.

## 2 Results and Discussion

### 2.1 Optimal Magnesium Concentration for Enhancing Biocompatibility and Neurite Outgrowth in PC12 Cells

**Figure 2:**
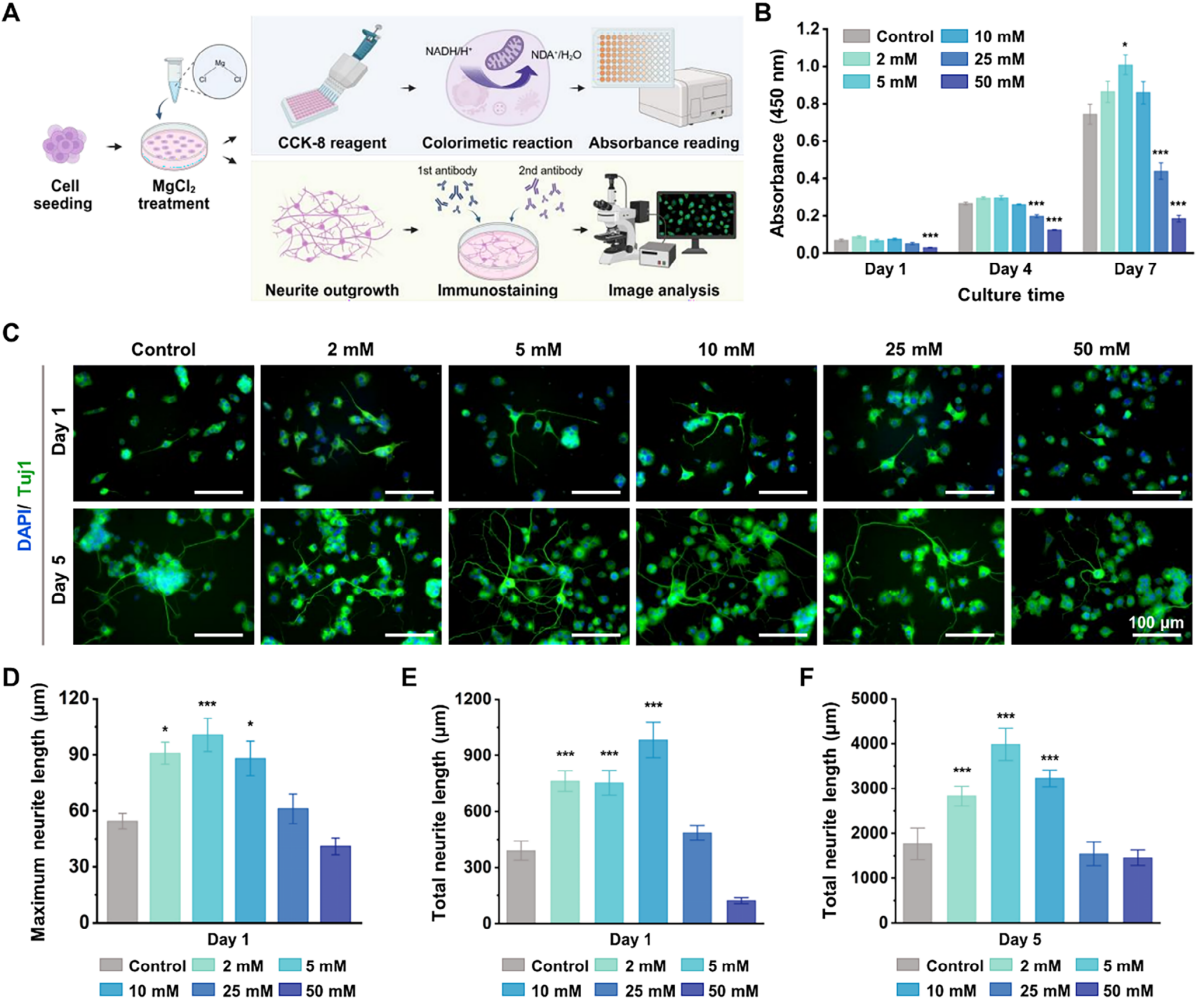
Optimal magnesium concentration for enhancing biocompatibility and neurite outgrowth in PC12 cells. A) Schematic of the experimental procedure for assessing biocompatibility and neurite outgrowth. B) The effects of different concentrations of Mg^2+^ on the viability of PC12 cells over 7 days (*n* = 5). C) Neurite outgrowth analysis in PC12 cells at day 1 and day 5 post‐treatment with different MgCl_2_ concentrations (0, 2, 5, 10, 25, and 50 mm). Tuj1 (green) visualizes neurites and DAPI (blue) for stains nuclei. (scale bars = 100 µm) D) Maximum neurite length at day 1. E,F) Total neurite length at days 1 and 5. Data are presented as mean ± SEM, with *p < 0.05 and ***p < 0.001 indicating significance compared to the control group, as determined by ANOVA followed Tukey's test.

The biocompatibility and neurite outgrowth of neuronal cells are critical indicators of successful nerve regeneration.^[^ ^32^ ^]^ To evaluate the bioactive properties of Mg ions, different concentrations were analyzed using PC12 cells (**Figure** **2**A). By day 7, the group treated with 10 mm Mg^2+^ exhibited significantly higher cell viability than the control group, indicating non‐toxicity and enhanced proliferation (Figure 2B). Additionally, we evaluated the neurite outgrowth within the same concentration range of Mg^2+^. Concentrations of 10 mm or lower resulted in a substantial increase in neurite outgrowth, with marked elongation of neurites and extensive network formation observed by day 5 (Figure 2C). Quantitatively, compared to the control group, treatment with 10 mm Mg^2+^ ions resulted in a 1.62‐fold increase in maximum neurite length (Figure 2D) and a 2.52‐fold increase in total neurite length (Figure 2E) on day 1. Furthermore, by day 5, total neurite length had increased 1.83‐fold (Figure 2F). Early neurite process formation was notably observed during the initial stages of differentiation, indicating the potential of Mg^2+^ to promote early nerve regeneration.^[^ ^33^ ^]^ These results are consistent with previous studies, which have established Mg acts as a biofunctional ion and a biochemical cue that facilitates neuronal development.^[^ ^34^, ^35^ ^]^ However, concentrations exceeding 25 mm failed to support proliferation or neurite outgrowth, suggesting that the effect of Mg is concentration‐dependent. This underscores the importance of developing nerve conduits capable of optimal concentration of Mg^2+^ to promote peripheral nerve regeneration.

### 2.2 Fabrication and Surface Characterization of Magnesium Sputtering on Collagen Sheet

**Figure 3:**
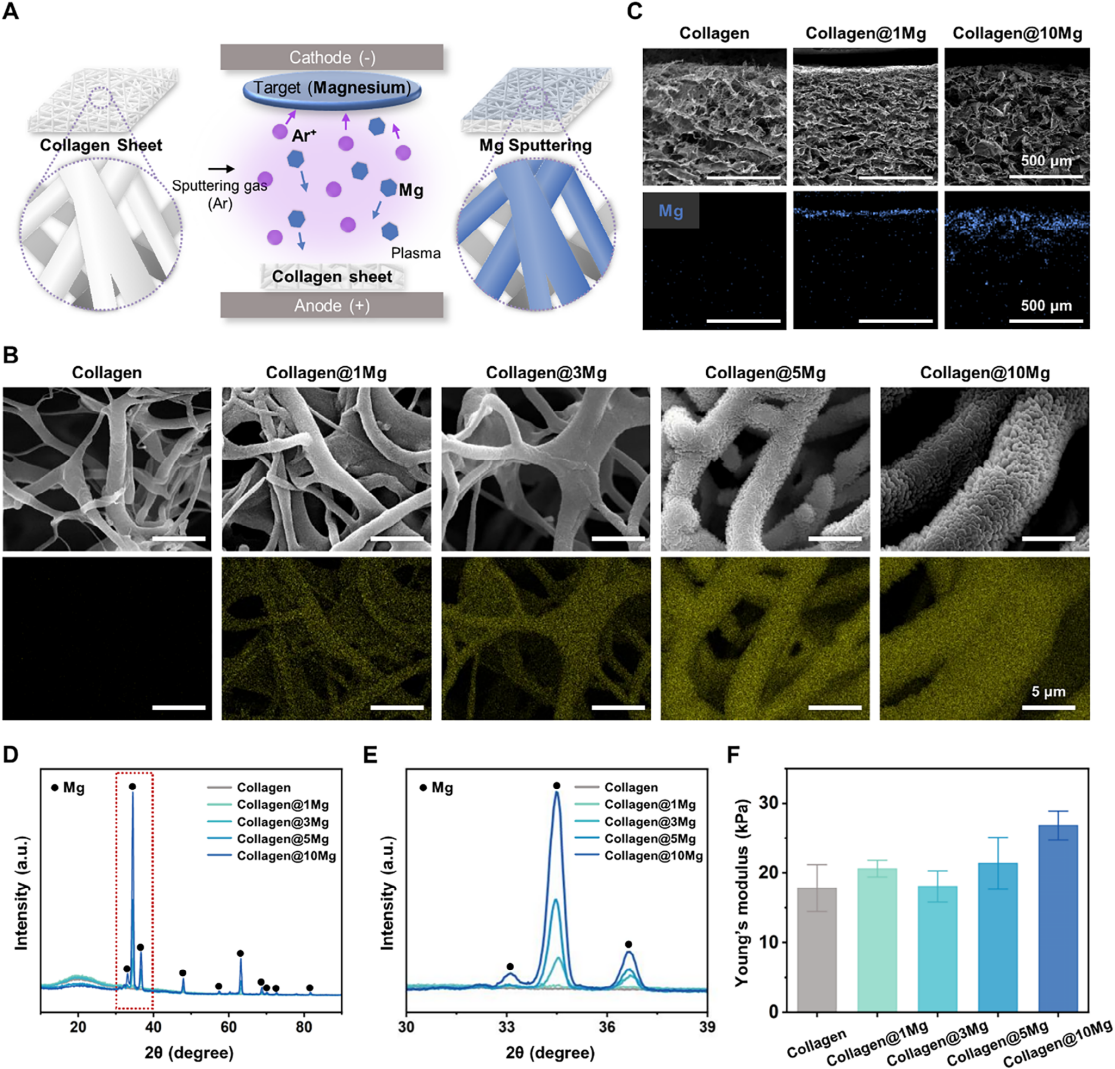
Fabrication, Characterization, and mechanical properties of Mg‐sputtered collagen sheet. A) Schematic illustration of the magnesium sputtering process on a collagen sheet. B) Elemental distribution analysis on the surface of collagen and Mg‐sputtered collagen sheets using EDS mapping (scale bar = 5 µm). C) Cross‐sectional SEM images and EDS maps showing the penetration and distribution of Mg in collagen and Mg‐sputtered collagen sheets. D) X‐ray diffraction (XRD) phase analysis of collagen and Mg‐sputtered collagen sheets. E) Magnified view of the XRD analysis between 30° and 39°. F) Young's modulus of collagen and Mg‐sputtered collagen sheets (*n* = 3). Data are presented as mean ± SEM.

Square‐shaped collagen sheets (10 mm × 10 mm) were prepared for fabrication and surface characterization, employing sputtering to achieve controlled Mg^2+^ delivery. During the process, collagen sheets were placed in a vacuum chamber where argon gas was introduced, creating plasma under DC magnetron sputtering conditions. Argon ions bombarded a pure Mg target, causing Mg atoms to sputter and deposit onto the collagen sheet (**Figure** **3**A). Surface verification of both bare and Mg‐sputtered collagen sheets was verified using optical imaging (Figure S1, Supporting Information). Macroscopically, the bare collagen sheet appeared opaque and white, while the Mg‐sputtered sheet exhibited a distinct gray appearance. Both surfaces remained well‐preserved without noticeable changes, indicating successful Mg deposition. EDS mapping analysis and SEM imaging were used to examine the surface morphology, microstructure integrity, and elemental distribution. Previous studies have demonstrated that collagen scaffolds maintain structural stability even after exposure to vacuum conditions, such as during freeze–drying or SEM imaging.^[^ ^36^, ^37^ ^]^ However, since the sputtering process involves prolonged vacuum exposure, verifying whether this condition could affect collagen fiber morphology was necessary. Our results confirmed that no significant differences in fiber morphology were observed before and after sputtering, indicating that the vacuum conditions did not induce shrinkage or distortion (Figure S2,Supporting Information). SEM images of the bare collagen sheet revealed a porous structure with an intact fibrous network, while the Mg‐sputtered sheet exhibited Mg particles uniformly accumulated on the fibers without deforming the network (Figure S3, Supporting Information). The Mg particles deposited on the collagen surface showed a hexagonal morphology, which is consistent with previous studies.^[^ ^38^ ^]^ Quantitative analysis of fiber thickness showed that increasing Mg sputtering resulted in greater fiber thickness (Figure S4, Supporting Information). EDS mapping demonstrated an increased intensity and uniform distribution of Mg on the fibers, which correlated positively with the number of sputtering cycles (Figure 3B). Cross‐sectional SEM images and corresponding EDS maps of collagen and Mg‐sputtered collagen sheets revealed that Mg deposition primarily occurred on the top surface of the collagen sheet, with the concentration decreasing gradually with depth. Increased sputtering cycles resulted in greater Mg penetration depth and thicker deposition layers while maintaining the microstructure integrity of the collagen sheets (Figure 3C). XRD surface analysis confirmed the material deposited by sputtering was Mg. The bare collagen sheets showed broad and undetectable XRD peaks, consistent with the low crystallinity of the polymer (Figure 3D).^[^ ^39^ ^]^ In contrast, distinct Mg peaks were observed on the Mg‐sputtered sheets, with no additional peaks corresponding to other elements or compounds.^[^ ^40^ ^]^ A magnified XRD analysis between 30° and 39° revealed that the intensity of Mg peaks increased progressively with increasing sputtering cycles, indicating an improved crystalline arrangement of Mg on the collagen surface (Figure 3E).

Sputtering technology has been widely utilized to enhance the bioactive properties of medical devices, such as biocompatibility, biological performance, and corrosion resistance, particularly in high‐durability materials like stainless steel and titanium‐based implants.^[^ ^41^ ^]^ However, the high‐speed ion impact inherent to the sputtering process can pose physical damage to the substrate and compromise its structural integrity. These issues are particularly pronounced when applied to sensitive biomaterials like collagen fibers, potentially causing structural distortion or the introduction of contaminants.^[^ ^42^ ^]^ To overcome these challenges, we optimized sputtering process parameters to preserve the structural integrity of collagen fibers, preventing distortion and avoiding contamination. This innovation presents a safe and effective method for delivering biofunctional magnesium ions to sensitive substrates.

### 2.3 Analysis of the Mechanical Properties of Mg‐Sputtered Collagen Sheet

Tensile testing was performed under dry conditions to evaluate the mechanical compatibility of Mg‐sputtered collagen sheets with peripheral nerve tissue. Previous studies have also conducted tensile tests under dry conditions for consistency and comparability.^[^ ^43^, ^44^ ^]^ The stress–strain (S–S) curve, depicting the relationship between applied stress and resulting strain (Figure S5, Supporting Information), showed a slight increase in the Young's modulus for the Mg‐sputtered collagen sheets (collagen@10Mg group: 26.79 ± 2.08 kPa) compared to bare collagen sheets (17.83 ± 3.37 kPa) (average ± SE, n = 3) (Figure 3F). According to Figure S4 (Supporting Information), Sputtering Mg onto soft collagen fibers resulted in a more compact fiber morphology with increased fiber thickness, which could have contributed to increased inter‐fiber friction and improved tensile strength. Despite this, the Young's modulus of the collagen@10Mg sheets showed no significant increase compared to pure collagen sheets. This is because the thin Mg layer deposited during sputtering had a minimal impact on the inherent mechanical properties of collagen.

Previous studies have reported that Mg conduits and filaments have been shown to provide mechanical support by preventing surrounding tissues from obstructing the nerve regeneration pathway.^[^ ^5^, ^26^ ^]^ The GDNF‐gel/HA‐Mg conduit could create a stable regenerative environment and enhance functional recovery in nerve injury models.^[^ ^26^ ^]^ However, despite these advantages, the high mechanical stiffness of Mg could lead to mechanical mismatch with nerve tissues, which may impose excessive mechanical stress on regenerating axons and potentially exacerbate nerve damage. On the other hand, Mg filaments are embedded within PCL nerve conduits, resulting in a reduced direct contact area with nerve cells compared to Mg conduits. Hopkins et al. reported that Mg filaments were found to mitigate inflammatory responses and promote short‐gap sciatic nerve regeneration in a transaction model.^[^ ^16^ ^]^ Tatu et al. demonstrated that plasma electrolytic oxidation (PEO) coating was applied to regulate the degradation rate of Mg filaments, facilitating axonal growth in a 15 mm long‐gap injury model.^[^ ^5^ ^]^ However, this approach did not lead to functional recovery. The Young's modulus of Mg, which is generally reported to be ≈45 GPa, may lead to mechanical mismatch with nerve tissue, potentially causing tissue damage.^[^ ^45^, ^46^ ^]^ In contrast, our approach prioritizes developing a soft nerve conduit that closely mimics the natural properties of peripheral nerve tissue (≈100 kPa).^[^ ^28^ ^]^ This soft conduit protects against secondary mechanical damage, soft tissue hyperplasia, and immunologic rejection and provides a suitable environment for neurite extension and Schwann cell proliferation and migration.^[^ ^47^ ^]^ By addressing mechanical mismatch issues, our Mg‐sputtered collagen sheets demonstrate significant advantages in creating a biocompatible and mechanically compatible nerve conduit.

Additionally, in vivo environments maintain collagen in a hydrated state. As hydration progresses, water molecules intercalate between collagen fibrils, weakening hydrogen bonding and other intermolecular interactions. These changes increase the softness of collagen, as reported in previous studies.^[^ ^48^, ^49^ ^]^ Considering this, we hypothesized that our Mg‐sputtered collagen sheet would exhibit better compatibility with nerve tissue under hydrated conditions. However, preliminary experiments revealed that the increased softness of hydrated collagen made it prone to tearing during tensile testing, complicating reliable mechanical measurements. To ensure precision and reproducibility, experiments were conducted under dry conditions. Nevertheless, we recognize the importance of physiologically relevant conditions and plan to refine our experimental setup for future tensile testing under hydrated conditions.^[^ ^50^ ^]^

### 2.4 Controllable Degradation in Mg‐Sputtered Collagen Sheets

**Figure 4:**
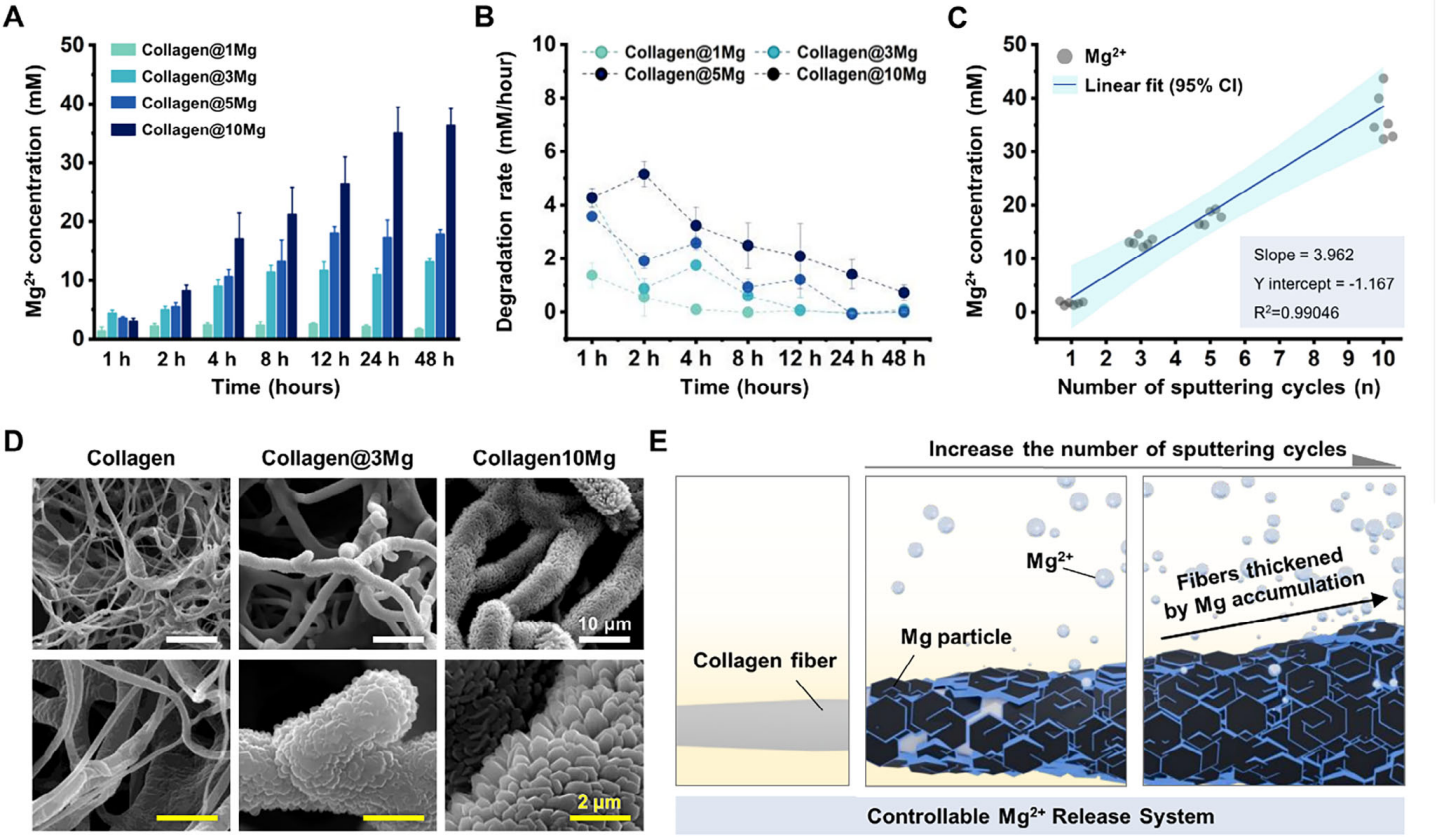
Controllable degradation in Mg‐sputtered collagen sheets. A) Mg concentration released from Mg‐sputtered collagen sheets over time (*n* = 3) B) The degradation rate of magnesium from collagen and Mg‐sputtered collagen sheets over time. C) Relationship between sputtering time and magnesium concentration in Mg‐collagen sheets. The blue line represents the linear fit, while the light blue shaded area around the regression line indicates the 95% confidence interval (CI). D) SEM images of collagen and Mg‐sputtered collagen sheets. (White scale bar = 10 µm, yellow scale bar = 2 µm) E) A Schematic diagram of the mechanism of a controllable Mg^2+^ release system. Data are presented as mean ± SEM.

We monitored Mg^2+^ release from the 1Mg, 3Mg, 5Mg, and 10Mg samples over time to confirm that the Mg‐sputtered sheets released an optimal concentration of Mg^2+^ suitable for promoting nerve regeneration. Previous experiments indicated that a 10 mm concentration of Mg^2+^ effectively induced neurite outgrowth. Among the samples, the collagen@3Mg sheet achieved a similar concentration (12.862 ± 0.250 mm) within 48 h, closely approximating the optimum Mg^2+^ concentration required for biocompatibility and neurite outgrowth in vitro (**Figure** **4**A). Additionally, the collagen@3Mg sheet exhibited a stable degradation profile, with the highest release rate recorded at 4.266 ± 0.328 mm within the first hour. This controlled release prevented rapid degradation, ensuring sustained therapeutic Mg^2+^ delivery over time (Figure 4B). Based on these results, the collagen@3Mg sheet was selected for subsequent experiments due to its ability to maintain a consistent and therapeutic Mg^2+^ concentration.

We demonstrated precise control over Mg^2+^ release by adjusting the number of sputtering cycles, as indicated by the strong linear correlation (R^2^ = 0.99046) (Figure 4C). This controllable degradation enhances the biocompatibility and biofunctionality of biomaterials while offering potential applications in tissue engineering. Furthermore, this approach mitigates issues such as excessive Mg accumulation, hydrogen gas pockets, and localized alkalization, which could otherwise impair the healing process.^[^ ^51^, ^52^ ^]^

The mechanism underlying this controlled degradation involves structural changes on the collagen surface. Initially, the collagen fibers exhibit a porous network. With increasing sputtering cycles, Mg deposition causes individual fibers to thicken, forming larger fibers (Figure 4D). As fiber thickness increases, the surface area‐to‐volume ratio decreases,^[^ ^53^ ^]^ reducing the total exposed Mg surface available for reaction with water.^[^ ^54^ ^]^ Consequently, the rate of Mg^2+^ release is effectively regulated, slowing degradation and mitigating corrosion. A schematic representation of this mechanism is provided in Figure 4E, illustrating how controllable Mg^2+^ concentrations and degradation rates can be achieved.

One of the major challenges in Mg‐based nerve conduits is their rapid corrosion and difficulty in controlling Mg release concentrations.^[^ ^55^ ^]^ Bhat et al. developed an Mg–1.6wt.%Li thin film to mitigate the rapid corrosion of Mg.^[^ ^20^ ^]^ It was observed that while the degradation rate of Mg–1.6wt.%Li was slower than that of pure Mg films, it released 20 mm of Mg^2+^ over 48 h. However, prolonged degradation could potentially lead to cytotoxicity. Yao et al. utilized a hyaluronic acid (HA)‐pamidronate (Pam)‐Mg hydrogel, which released ≈4 mm of Mg^2+^ over 15 days,^[^ ^56^ ^]^ and embedded MgO/MgCO_3_ into PCL,^[^ ^57^ ^]^ which achieved a total release of 5 mm of Mg^2+^ over 8 weeks, promoting nerve regeneration. However, the daily Mg^2+^ release rates in these studies were 0.27 and 0.09 mm, respectively, which are significantly lower than the optimal concentration of 10 mm reported for nerve regeneration. ^[^ ^56^, ^57^ ^]^ In this study, the Mg‐sputtered collagen sheet was developed to precisely regulate Mg release by adjusting sputtering cycles. This approach effectively addresses the limitations of existing strategies, such as rapid corrosion and uncontrolled Mg concentrations, thereby creating an optimal Mg release for nerve regeneration. However, while this technology successfully suppresses rapid corrosion and regulates release concentrations, it has limitations in maintaining sustained Mg release over extended periods. Future research will explore sputtering Mg alloys or additional coating technologies to ensure a continuous and optimal Mg release profile.

### 2.5 Biocompatibility and Neurite Outgrowth Induced by Collagen@3Mg

The potential toxicity and differentiation effects of collagen and collagen@3Mg extracts on PC12 cells were evaluated. Viability assay results demonstrated a significant improvement (**p < 0.01) in cell viability for both collagen and collagen@3Mg extract treatments compared to the media alone after 7 days of culture (Figure S6, Supporting Information).

Phase‐contrast images and immunofluorescent staining revealed substantial neurite outgrowth and neural network formation after 5 days of treatment with collagen@3Mg extracts (Figure S7, Supporting Information). In contrast, media and collagen treatment did not significantly promote neuronal growth. Quantitatively, the total neurite length induced by collagen@3Mg extracts was 2.34‐ and 1.91‐fold higher than that observed with media and collagen extracts, respectively (***p < 0.001) (Figure S8, Supporting Information). The controlled Mg^2+^ release from collagen@3Mg exhibited enhanced biocompatibility and neurite outgrowth promotion, aligning with the previously observed effects of MgCl_2_ (Figure 2). These findings suggest that collagen@3Mg is a promising candidate for application in peripheral nerve regeneration.

### 2.6 Early Nerve Regeneration in CPL@3Mg

**Figure 5:**
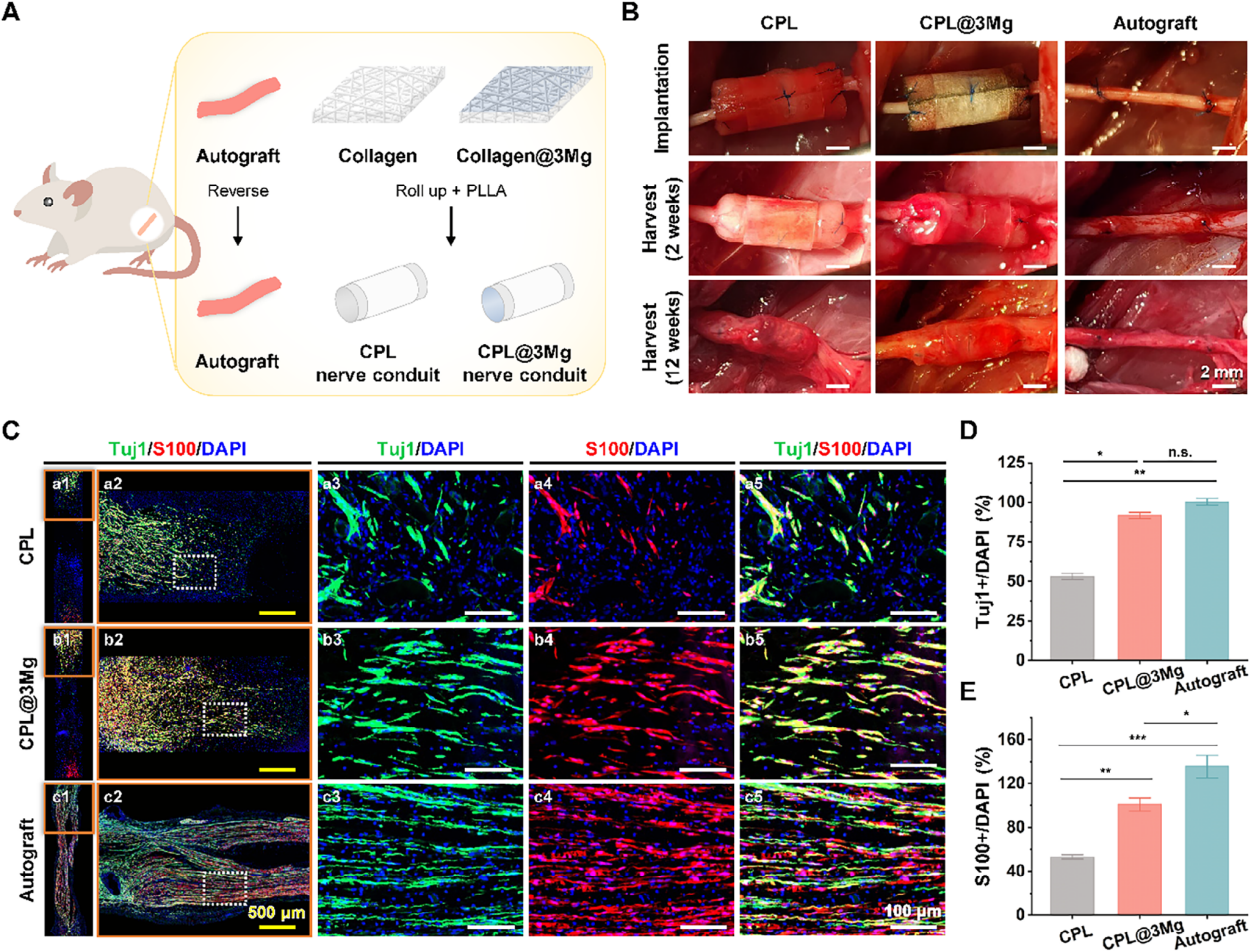
Enhanced early nerve regeneration in Mg‐sputtered collagen sheets. A) Schematic of the in vivo experimental design for sciatic nerve repair in rats. B) Implantation and 2‐week post‐operation images of nerve grafts in rat sciatic nerve model (scale bar = 2 mm). C) Immunofluorescence staining images of longitudinal sections of the grafts, showing axons (green), Schwann cells (red), and nuclei (blue). The orange solid line indicates an enlarged view of the proximal part of the longitudinal section. The white dotted line shows a highly magnified view of a specific area within the proximal part (scale bar = 100 µm). D,E) The quantitative analysis compared to the CPL, CPL@3Mg, and autograft group. Data are presented as mean ± SEM, with *p < 0.05, **p < 0.01, and ***p < 0.001 indicating significance compared to the control, as determined by Tukey's test, n.s. = not significant.

The effects of Mg‐sputtered collagen nerve conduit on nerve regeneration were assessed using a rat defect model. The autograft, CPL, and CPL@3Mg nerve conduits were transplanted into 10 mm sciatic nerve defect sites. The proximal and distal nerve ends were inverted and sutured in the autograft group. In the CPL and CPL@3Mg groups, nerve conduits were constructed by rolling bare collagen or Mg‐sputtered collagen sheets into cylindrical shapes, and the structure was stabilized by wrapping the conduits in PLLA (**Figure** **5**A). All animals underwent successful surgery without adverse effects, including hydrogen gas pocket formation, which is often associated with rapid Mg degradation (Figure 5B).

Histological assessments were conducted 2 weeks post‐transplantation to investigate early neurogenesis and the therapeutic effects of the conduits. Immunofluorescence staining with Tuj1 (axon marker) and S100 (Schwann cell marker) was used to evaluate axonal and Schwann cell regeneration (Figure 5C). In the autograft group, the nerve defect site was fully bridged from the proximal to the distal end. However, in the CPL and CPL@3Mg groups, full nerve connection was not achieved within the 2 weeks. This outcome is consistent with previous studies, which have shown that attaining complete nerve regeneration within 2 weeks using nerve conduit is highly challenging.^[^ ^58^, ^59^ ^]^ In the proximal region (highlighted by the orange solid line), where axon regeneration is most active, a substantial increase in Tuj1– and S100– positive cells were observed in the CPL@3Mg group compared to the CPL group. Similarly, in regions marked by white dotted squares, the autograft group exhibited the highest expression of Tuj1 and S100, while the CPL@3Mg group demonstrated significantly higher expression levels than the CPL group. This trend was confirmed by quantitative analysis (Figure 5D,E). Although the autograft group exhibited the highest regeneration, Tuj1 expression in the CPL@3Mg group was similar to that in the autograft group. Furthermore, the CPL@3Mg group showed a significantly higher percentage of Tuj1– and S100– positive cells than the CPL group.

Although numerous studies have investigated the neuro‐regenerative potential of Mg, few have reported significant nerve regeneration within the first 2 weeks post‐injury. Most prior studies have primarily focused on long‐term regenerative outcomes, proposing various mechanisms through which Mg facilitates nerve repair. For instance, GDNF‐Gel/HA‐Mg conduits have been shown to promote peripheral nerve repair by activating peroxisome proliferator‐activated receptor gamma (PPAR‐γ) and inhibiting the Ras homolog family member A (RhoA)/Rho‐associated coiled‐coil containing protein kinase (ROCK) signaling pathway.^[^ ^26^ ^]^ Additionally, Li–Mg–Si ceramics have been reported to upregulate neurotrophic factor expression in a β‐catenin‐dependent manner.^[^ ^21^ ^]^ Moreover, MgO/MgCO₃/PCL multi‐gradient fibers were found to facilitate peripheral nerve regeneration by modulating Schwann cell function and activating the Wnt signaling pathway.^[^ ^57^ ^]^ However, few studies have reported significant nerve regeneration within the first 2 weeks post‐injury despite these advances. In contrast, our study confirmed that CPL@3Mg significantly promoted nerve regeneration within just 2 weeks, suggesting that precise regulation of Mg release can dramatically accelerate nerve regeneration. These findings highlight the potential of CPL@3Mg as a superior Mg‐based strategy, offering a more effective approach to enhancing early nerve repair.

### 2.7 Functional Analysis in CPL@3Mg

**Figure 6:**
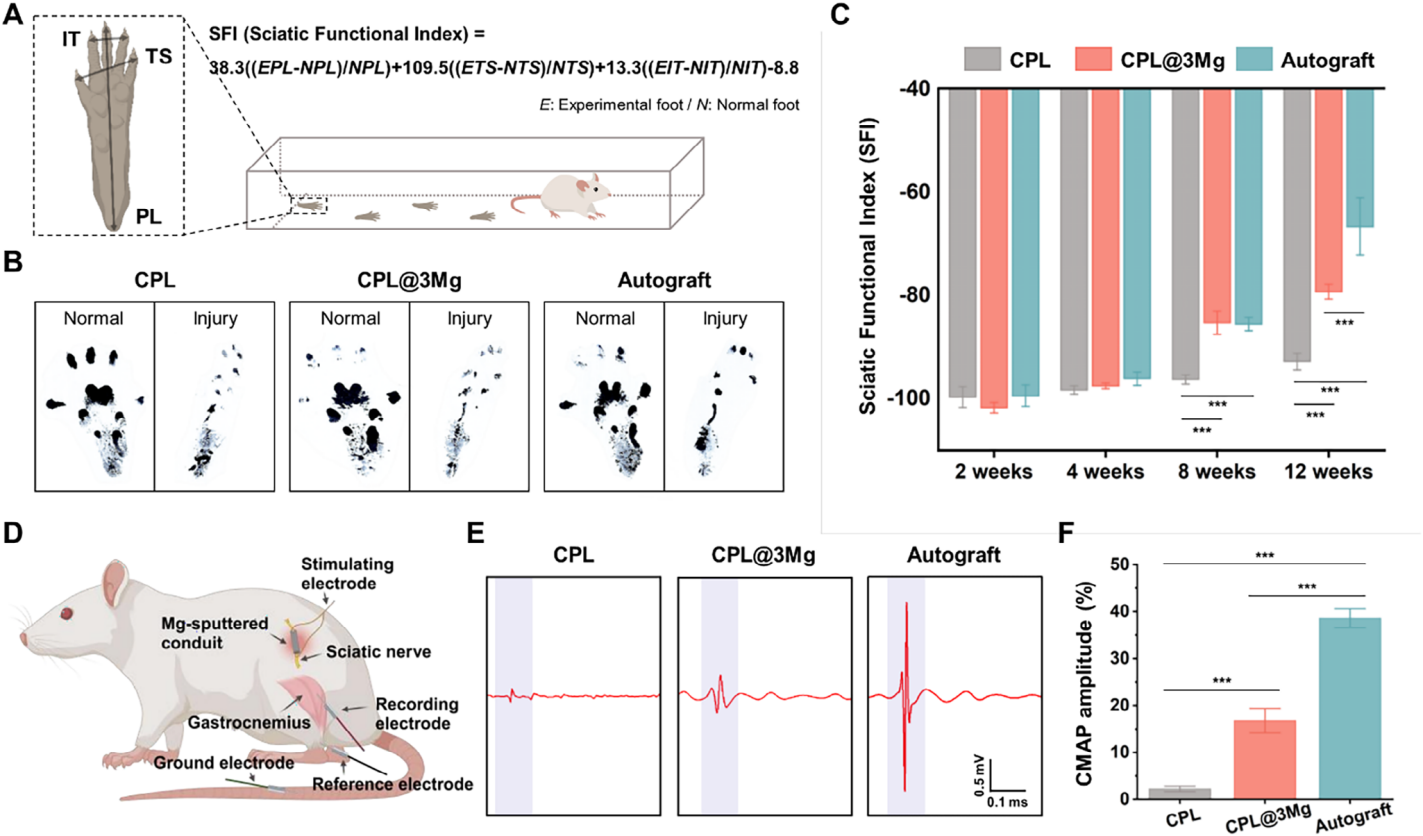
Functional recovery analysis via walk analysis and electrophysiological assessment. A) Schematic illustration of the SFI analysis (E, Experimental foot; N, Normal foot; IT, intermediate toe; TS, Toe spread; PL, Print length). B) Representative images of rat footprints in each group at 12 weeks. C) SFI values of each group at post‐operative time points. One‐way ANOVA followed by Tukey's post hoc test was used for statistical analysis, with comparisons to the CPL group. D) Schematic for the CMAP recording setup. E) Representative CMAP patterns from each group after 12 weeks. F) Quantitative analysis of CMAP amplitudes at 12 weeks. Data are presented as mean ± SEM, with ***p < 0.001 indicating significance between different groups, as determined by ANOVA followed by Tukey's test.

Walking and electrophysiological measurements were conducted to evaluate functional recovery.^[^ ^60^, ^61^ ^]^ The sciatic functional index (SFI) was calculated using footprint parameters (*IT*, *TS*, *PL*) and the SFI function formula (**Figure** **6**A). Representative footprints from each group revealed distinct differences in functional recovery, with more normal footprints observed in the CPL@3Mg and autograft groups compared to the CPL group (Figure 6B). SFI values improved across all groups, with the autograft group exhibiting the most significant recovery. However, the CPL@3Mg group demonstrated notably superior functional recovery at 8 and 12 weeks compared to the CPL group (***p < 0.001) (Figure 6C). Electrophysiological analysis corroborated these findings, showing significantly higher compound muscle action potential (CMAP) amplitudes in the CPL@3Mg and autograft groups compared to the CPL group (Figure 6D,F) (***p < 0.001). In addition to enhanced early nerve regeneration, functional recovery, as assessed through walking and electrophysiological analysis, significantly improved in the CPL@3Mg group compared to the CPL group. This finding underscores the critical advantage of CPL@3Mg, as its benefits extend beyond tissue recovery to include the restoration of nerve function. The improvement in functional recovery is a critical factor in the success of nerve regeneration strategies,^[^ ^62^ ^]^ highlighting that CPL@3Mg plays a key role not only in cellular regeneration but also in restoring nerve function.

### 2.8 Functional Recovery of Reinnervated Muscles in CPL@3Mg

Reinnervation of the gastrocnemius muscle by regenerating nerve fibers facilitates improved muscle recovery and reduced atrophy. Twelve weeks postoperatively, the gastrocnemius muscles were analyzed, and the wet weight ratios in the CPL and CPL@3Mg groups were lower than those in the autograft group (Figures S9 and S10, Supporting Information).

**Figure 7:**
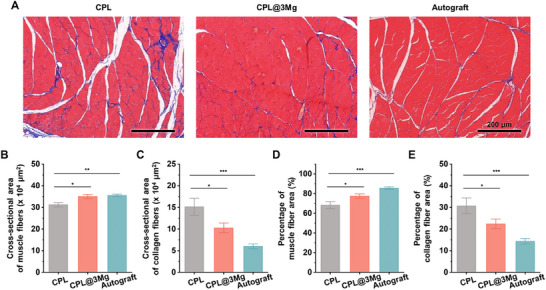
Functional recovery of reinnervated muscles. A) Masson's trichrome staining of transverse sections of the gastrocnemius muscles at 12 weeks. B–E) Quantitative analysis of the cross‐sectional area of muscle and collagen fibers (µm^2^) and percentage area of muscle and collagen fibers. Data are presented as mean ± SEM, with *p < 0.05, **<0.01, and ***p < 0.001 indicating significance between groups, as determined by ANOVA followed Tukey's test.

Histological analysis of the cross‐sections stained with Masson's trichrome provided insights into muscle structure and fibrosis (**Figure** **7**A). The cross‐sectional area of the muscle fibers was significantly larger in the autograft and CPL@3Mg groups compared to the CPL group (*p < 0.05, **p < 0.01), with no significant difference between the autograft and CPL@3Mg groups. These findings suggest that muscle recovery in the CPL@3Mg group was comparable to that in the autograft group (Figure 7B). The cross‐sectional area of the collagen fibers was significantly lower in the CPL@3Mg and autograft groups compared to the CPL group (*p < 0.05, ***p < 0.001), with no significant difference between CPL@3Mg and autograft groups. This reduction in collagen deposition in the CPL@3Mg group to autograft levels indicates minimized fibrosis and better preservation of muscle integrity (Figure 7C). The muscle and collagen fiber area percentages showed a similar trend (Figure 7D,E). The CPL@3Mg group reduced muscle atrophy minimized fibrosis and achieved reinnervation and muscle recovery nearly equivalent to autografts, underscoring its effectiveness in promoting muscle reinnervation.

### 2.9 Nerve Regeneration in CPL@3Mg

**Figure 8:**
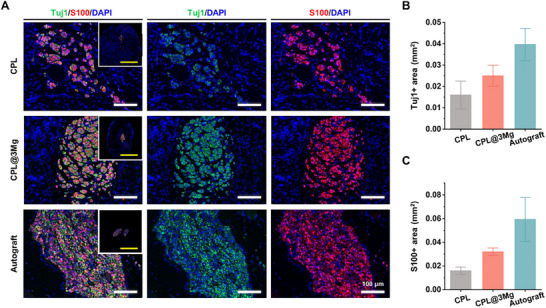
Enhanced neural regeneration in Mg‐sputtered collagen sheets. Immunofluorescence staining of cross‐sections of the grafts showing Tuj1 (green, axon marker), S100 (red, Schwann cells marker), DAPI (blue, nuclear) in the CPL, CPL@3Mg, and autograft groups at 12 weeks. (Yellow scale bar = 1 mm, white scale bar = 100 µm) B) Quantitative analysis of the Tuj1‐positive area (mm^2^) indicating axonal regeneration C) Quantitative analysis of the S100‐positive area (mm^2^) indicating Schwann cell presence. Data are presented as mean ± SEM. Statistical significance was determined using Tukey's post‐hoc test.

To evaluate nerve regeneration, we performed histological assessments of the middle portion of the nerve samples harvested at 12 weeks postoperatively. Immunofluorescence staining was used to visualize nerve fibers and nerve fascicle regeneration in the CPL@3Mg group (**Figure** **8**A). The autograft group exhibited the highest areas of Tuj1 (an axonal regeneration marker) and S100 (a Schwann cell marker), followed by the CPL@3Mg group and CPL group. However, no statistically significant differences were observed among the groups (Figure 8B,C), indicating that all three groups achieved histological recovery upon long‐term assessment.

Overall, CPL@3Mg exhibits substantial potential as a viable alternative to autograft. While previous studies on Mg‐based nerve regeneration have primarily focused on filaments,^[^ ^5^, ^63^, ^64^ ^]^ alloys,^[^ ^19^, ^20^, ^65^ ^]^ and hydrogels,^[^ ^56^, ^58^, ^66^ ^]^ this study introduces an innovative strategy by sputtering Mg onto a soft collagen sheet. Future studies should explore the potential synergistic effects of incorporating other biocompatible metal ions or investigate methods to enhance nerve regeneration through electrical stimulation, further combining multiple strategies.^[^ ^70^ ^]^

## 3 Conclusion

This study introduces a controllable degradation system for Mg‐based biomaterials using sputtering technology, marking a significant advancement in nerve regeneration research. Mg‐sputtered nerve conduits demonstrated enhanced biocompatibility, biofunctionality, mechanical compatibility, and precise magnesium release, resulting in improved axonal regeneration and functional recovery. These findings underscore the potential of CPL@3Mg conduits as a superior alternative to traditional autografts, offering an innovative and effective solution for peripheral nerve repair. This approach contributes significantly to the field of neural tissue engineering, providing a robust platform for advancing nerve regeneration therapies. Future research should focus on leveraging Mg alloys to improve further biosafety and biofunctionality, as well as exploring the conductive properties of Mg for electrical stimulation for improved peripheral nerve regeneration.

## 4 Experimental Section

### PC12 Cell Culture

PC12 cells were purchased from the American Type Culture Collection (ATCC, CRL‐1721). The proliferation medium consisted of RPMI supplemented with 10% horse serum, 5% fetal bovine serum, and 1% penicillin–streptomycin. The cells were cultured in an incubator at 37 °C with 95% humidity and 5% CO_2_. For differentiation, PC12 cells were seeded in a 24‐well plate coated with collagen (20 µg cm^−2^, Corning) and cultured in differentiation media with 1% horse serum, 1% penicillin–streptomycin, and 50 ng mL^−1^ nerve growth factor (NGF, Allomone Labs).

### Biocompatibility and Axon Regeneration in PC12 Cells

To access cell viability, PC12 cells (5000 cells per well) were seeded in a 96‐well plate and incubated for 1 day at 37 °C with 5% CO_2_ for cell stabilization. Thereafter, the medium was replaced with the experimental reagents to assess the optimal Mg^2+^ concentration and biocompatibility of the material. For Mg^2+^ concentration testing, MgCl_2_ (0, 2, 5, 10, 25, and 50 mm in proliferation media) was added for 2 days, with 0 mm MgCl_2_ serving as the control. For biocompatibility testing, nerve conduit sheet extracts were used as experimental samples. The cells were incubated for 1, 4, and 7 days. Cell viability was assessed using the CCK‐8 assay at each time point, and absorbance was measured at 450 nm using a microplate reader to determine the appropriate Mg^2+^ concentration.

Axonal regeneration was determined by culturing PC12 cells at a density of 8 × 10^4^ cells mL^−1^ at the 24‐well plate coated with 20 µg cm^−2^ collagen. After 24 h, the medium was replaced with experimental differentiation media containing different concentrations of MgCl_2_ (0, 2, 5, 10, 25, and 50 mm), with 0 mm MgCl_2_ serving as the control and nerve conduit sheet extracts. On days 1 and 5, cells were fixed in 4% paraformaldehyde for 15 min at room temperature, immersed in 0.25% Triton X‐100, blocked with 2% fetal bovine serum (FBS) and 0.5% Tween20, and incubated overnight at 4 °C with the anti‐beta III tubulin (Tuj1) antibody (1:200, D71G9, Cell Signaling Technology). Cells were treated with Alexa Fluor 488‐conjugated goat anti‐rabbit IgG (1:200, ab150077, Abcam) at room temperature for 2 h. Fluorescence was visualized using a mounting medium containing DAPI (H‐1200, Vector Laboratories, USA), and cells were analyzed with a fluorescence microscope (Imager A2 m, Zeiss, Germany). Neurite length was quantitatively measured using the IMARIS Filament Tracer module (version 9.1.2; Bitplane, an Oxford Instruments Company Inc).

### Fabrication of Magnesium Collagen Sheets

Collagen sheets were purchased from Hyundai Bioland, cross‐linked with 20 mm EDC (1‐ethyl‐3‐(3‐dimethylaminopropyl) carbodiimide hydrochloride), and subsequently lyophilized. Mg‐coated collagen sheets were fabricated using DC magnetron sputtering (Korea Vacuum Tech, KVS‐2000L). Collagen sheets (10 mm × 10 mm) were positioned in front of a 75 mm diameter, 10 mm thick Mg target (99.99% purity, VTM, Korea) inside a vacuum chamber evacuated to 1.6 × 10^−6^ Torr using rotary and diffusion pumps. A target power of 130 W was applied, maintaining a working pressure of 3 mTorr, to sputter Mg^2+^ and neutral atoms onto the collagen substrate for 30 min. Sputtering was performed in 30‐min cycles followed by 30 min of rest, with the number of cycles denoted as nMg.

### Characterization of Magnesium Collagen Sheet

Before imaging, samples were coated with a thin layer of platinum to enhance conductivity and contrast. The coated surfaces and cross‐sections were examined using a scanning electron microscope (SEM; Inspect50, FEI, USA) to assess the coating's morphology and structural properties. Fiber thickness was quantitatively analyzed from SEM images (*n* = 10 per group). Energy‐dispersive X‐ray spectroscopy (EDS) integrated with SEM was employed to analyze the distribution and composition of elements. X‐ray diffraction (XRD; D8 ADVANCE, Bruker, USA) with Cu Kα radiation was conducted to determine the phases and microstructures of the coating materials. Measurements were performed in the 2θ range of 10°–90° in intervals of 0.02°.

Tensile mechanical strength tests were conducted on the collagen and Mg‐sputtered collagen sheets under dry conditions. The sheets were cut into rectangular samples with dimensions of 10 mm × 50 mm, and the tests were conducted using a universal testing machine (F105, Mark‐10) at a tensile speed of 20 mm min^−1^. S–S curves were generated by measuring the applied force and corresponding elongation of the samples. The S–S curves represent representative data that reflect the overall trend of the dataset. Young's modulus was calculated within the linear region (a strain range of 3–17%), excluding the Toe region (nonlinear initial region) and the Failure region (fracture region).^[^ ^67^, ^68^ ^]^ Linear fitting was individually applied to the segment that met the criterion of R^2^ ≥ 0.99.

### Degradation and Mg Release

To evaluate the degradation profiles of the Mg‐sputtered collagen sheets, different sputtering cycles (1Mg, 3Mg, 5Mg, and 10Mg) were tested. The collagen sheets were immersed in RPMI medium supplemented with 1% horse serum and 1% penicillin–streptomycin to ensure consistent exposure conditions. The samples were incubated at 37 °C in a 5% CO_2_ environment to simulate human physiological conditions. RPMI medium samples containing eluted Mg^2+^ were collected after 1, 4, and 7 days. The concentration of Mg^2+^ was quantified using the Quantichrom Metal Ion Assay kit (DIMG‐250, Bioassay Systems, USA). The accumulated Mg^2+^ concentration and elution rate were measured, providing insights into the Mg‐sputtered collagen sheets' degradation behavior and release profiles.

### Surgical Preparation of In Vivo Model

All animal studies followed the principles for the care and use of laboratory animals, as approved by the Institutional Animal Care and Use Committee (IACUC) (Animal experiment ethics approval number: KIST‐IACUC‐000‐1). A total of 18 male Sprague Dawley rats (8 weeks old, 250–270 g) were purchased from Central Lab. Animal Inc. The rats were anesthetized with 2% inhaled isoflurane and placed on a 37 °C heating pad. The surgical site (lateral right thigh) was shaved and sterilized with 70% alcohol before making a 4 cm incision to expose the sciatic nerve. The rats were randomly divided into three groups: 1) autograft, 2) CPL, and 3) CPL@3Mg. In the autograft group, a 10 mm sciatic nerve was excised, and the proximal and distal ends were inverted and sutured for end‐to‐end neuroanastomosis using 7‐0 Prolene. In the CPL group, collagen sheets (10 mm length × 12 mm diameter) were rolled and sutured with 7‐0 Prolene, whereas in the CPL@3Mg group, the sheets were first sputtered with 3Mg and processed similarly. For both the CPL and CPL@3Mg groups, an 8 mm × 13 mm poly(l‐lactic acid) (PLLA) sheet was wrapped around the nerve conduits to maintain a cylindrical shape. The FDA‐approved PLLA sheet, known for its high biocompatibility, was manufactured shorter to prevent direct contact with the nerve. The conduits were transplanted via end‐to‐end anastomosis using 7‐0 Prolene, leaving a 10 mm gap. The skin was aseptically closed with 4‐0 Prolene. Gentamicin (5 mg kg^−1^) and ketoprofen (5 mg kg^−1^) were administered pre‐ and postoperatively to manage infection and pain. Group randomization was alternated during the operational window, and all experiments were conducted at a consistent time of day to minimize the effect of circadian rhythm effects.

### Functional Evaluation of Nerve Regeneration

The functional recovery of individual rats was assessed by walk analysis at 2, 4, 8, and 12 weeks postoperatively. Each rat was tested in a confined walkway (10 cm × 50 cm, covered with white paper) after being trained to traverse the walkway twice or thrice. During testing, distinct and clear footprints were obtained and analyzed to calculate the sciatic functional index (SFI). A pairwise comparison of three left and right footprints for each rat was used to determine the SFI. An SFI value of 0 indicates normal function, while an SFI of −100 represents a complete loss of function.^[^ ^69^ ^]^

Functional recovery of the regenerated nerves was further assessed through electrophysiology 12 weeks postoperatively. Each rat was anesthetized, and the left and right sciatic nerves were surgically re‐exposed. Electrical stimulation electrodes were applied to the sciatic nerve, and pulse signals were delivered (stimulus intensity, 400 mV; duration, 1 ms; Frequency, 1 Hz) using a function generator (AFG1062, Tektronix). Recording, reference, and ground electrodes were positioned on the gastrocnemius muscle belly, ankle tendon, and tail, respectively. A temperature‐controlled and low‐noise environment was maintained to minimize external interference during recordings. Compound muscle action potentials (CMAPs) were recorded using a LabRat system (LR10‐SK1, Tucker‐Davis Technologies). For each rat, three measurements were obtained for both the injured and contralateral sides, and the CMAP amplitudes were quantified by comparing these measurements.

### Evaluation of Reinnervated Muscle

Both experimental and control gastrocnemius muscles were harvested from each group at 12 weeks postoperatively. The wet weight ratio of the muscle was calculated by comparing the wet weight of the experimental muscle to the control muscles. Harvested muscles were fixed, embedded in paraffin, and cross‐sectioned for Masson's trichrome staining (KH07007, Bioquochem). Randomly selected fields of view were photographed for each specimen. The percentage of muscle fibers was calculated by dividing the muscle fiber area by the total area of the muscle and collagen fibers, as measured using ImageJ software.

### Histological Evaluation of Regenerated Nerves

Individual animals were euthanized at 2‐ and 12 weeks post‐surgery, and their nerves were harvested, fixed in 4% paraformaldehyde overnight, and embedded in paraffin blocks. For early nerve regeneration analysis, specimens from 2 weeks post‐surgery were longitudinally sectioned (5 µm thick) through the center of the nerve conduit using a microtome (CM3050S; Leica Microsystems). At 12 weeks, the middle region of the nerves was cross‐sectioned into 5 µm thick slices. After deparaffinization and rehydration, antigen retrieval was performed using a target retrieval solution. Primary antibodies included mouse anti‐beta‐tubulin (Tuj1) (1:200; T8578; Sigma–Aldrich) and rabbit anti‐S100 (1:200; ab52642; Abcam). The secondary antibodies used were anti‐mouse Alexa Fluor 488 (1:200; A21202; Invitrogen) and anti‐rabbit Alexa Fluor 594 (1:200; A21207; Invitrogen). Sections were incubated with diluted primary antibodies overnight at 4 °C, washed with PBS three times, and incubated with secondary antibodies for 90 min at room temperature in the dark. The sections were rewashed with PBS, stained with DAPI, and mounted on slides. The stained sections were visualized using a fluorescence microscope (Axioscan7, Zeiss, Germany). At 2 weeks, the areas of Tuj1, DAPI, and S100 immunoreactivity within the entire conduit were quantified. To normalize measurements to the total cell population, the areas of Tuj1 and S100 were divided by the corresponding DAPI areas. At 12 weeks, the Tuj1‐ and S100‐positive areas were quantified within the entire conduit. At this time point, the internal structure of the conduit was less distinct due to its integration with the surrounding native tissues. Therefore, DAPI was not normalized, as the conduit boundaries were no longer identifiable. Image analysis used ImageJ with consistent thresholding and segmentation parameters across all samples to ensure reproducibility. Additionally, images were analyzed under blinded conditions, ensuring the operators were unaware of the group assignments.

### Statistical Analyses

Statistical significance testing was conducted using numerical data from each experiment, and graphing and statistical analysis software (Origin, OriginLab Corporation) was employed. Data are presented as mean ± standard error of the mean (SEM). Statistical differences were determined using one‐way factorial analysis of variance (ANOVA) followed by Tukey's post hoc test, with a significance level set at *p* < 0.05.

## Conflict of Interest

The authors declare no conflict of interest.

## Supporting information



Supporting Information

## Data Availability

The data that support the findings of this study are available from the corresponding author upon reasonable request.
