# Supplementary material for: Controlled Magnesium Ion Delivery via Mg‐Sputtered Nerve Conduit for Enhancing Peripheral Nerve Regeneration
Source: Adv Healthc Mater. 2025 Apr 27;14(20):2500063. doi: 10.1002/adhm.202500063 (PMC12333477; doi:10.1002/adhm.202500063)
Supplement: Supplementary file 1 — Supporting Information [file ADHM-14-0-s001.docx]

Supporting Information

Controlled magnesium ion delivery via Mg-sputtered nerve conduit for enhancing peripheral nerve regeneration

*Hyewon Kim, Jieun Kwon, Hyeok Kim, Sunhee Lee, Seongchan Kim, Ji-Young Lee, Khandoker Asiqur Rahaman, Taeyeon Kim, Hyojin Lee, Myoung-Ryul Ok, Seok Chung*, Hyung-Seop Han*, Yu-Chan Kim**

H. Kim, J. Kwon, H. Kim, S. Lee, J.-Y. Lee, K. A. Rahaman, T. Kim, H. Lee, M.-R. Ok, H.-S. Han, Y.-C. Kim

Biomaterials Research Center

Biomedical Research Division

Korea Institute of Science and Technology (KIST)

Seoul 02792, Republic of Korea

E-mail: hyuhan@kist.re.kr, chany@kist.re.kr

H. Kim, S. Chung

Department of Biomicro System Technology

Korea University

Seoul 02841, Republic of Korea

E-mail: sidchung@korea.ac.kr

S. Kim

College of Pharmacy and Research Institute of Pharmaceutical Sciences

Gyeongsang National University

Jinju, Gyeongsangnam-do, 52828, Republic of Korea

J.-Y. Lee, M.-R. Ok, H.-S. Han, Y.-C. Kim

Division of Bio-Medical Science & Technology

KIST school

University of Science and Technology (UST)

Seoul 02792, Republic of Korea

H. Lee

SKKU-KIST, Department of Integrative Biotechnology, College of Biotechnology and

Bioengineering, Sungkyunkwan University, Gyeonggi, Suwon, 16419, South Korea


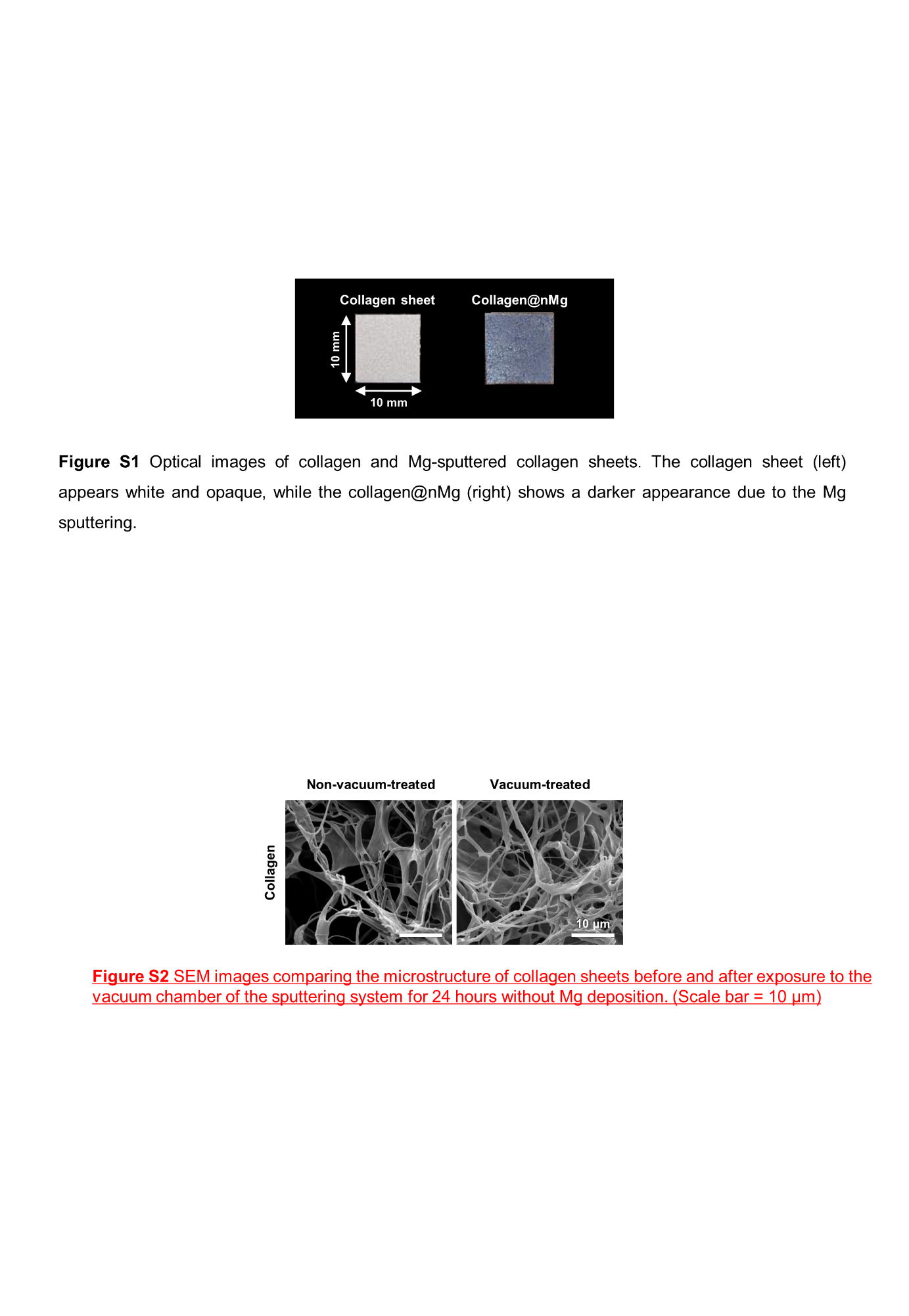


**Figure S1** Optical images of collagen and Mg-sputtered collagen sheets. The collagen sheet (left) appears white and opaque, while the Mg-sputtered collagen sheet (collagen@nMg, right) shows a darker appearance due to the Mg sputtering.


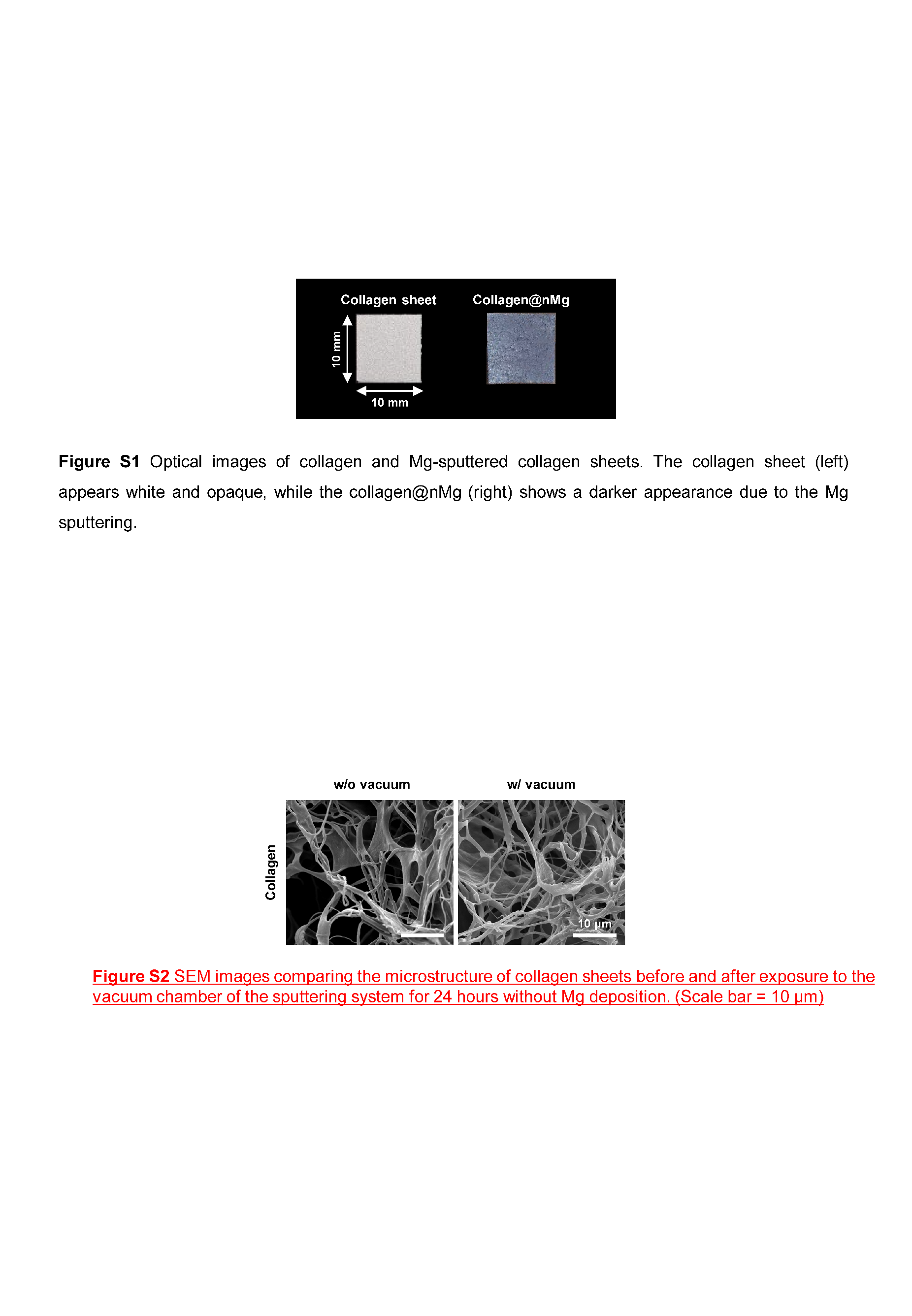


**Figure S2** SEM images comparing the microstructure of collagen sheets before and after exposure to the vacuum chamber of the sputtering system for 24 hours without Mg deposition. (Scale bar = 10 μm)


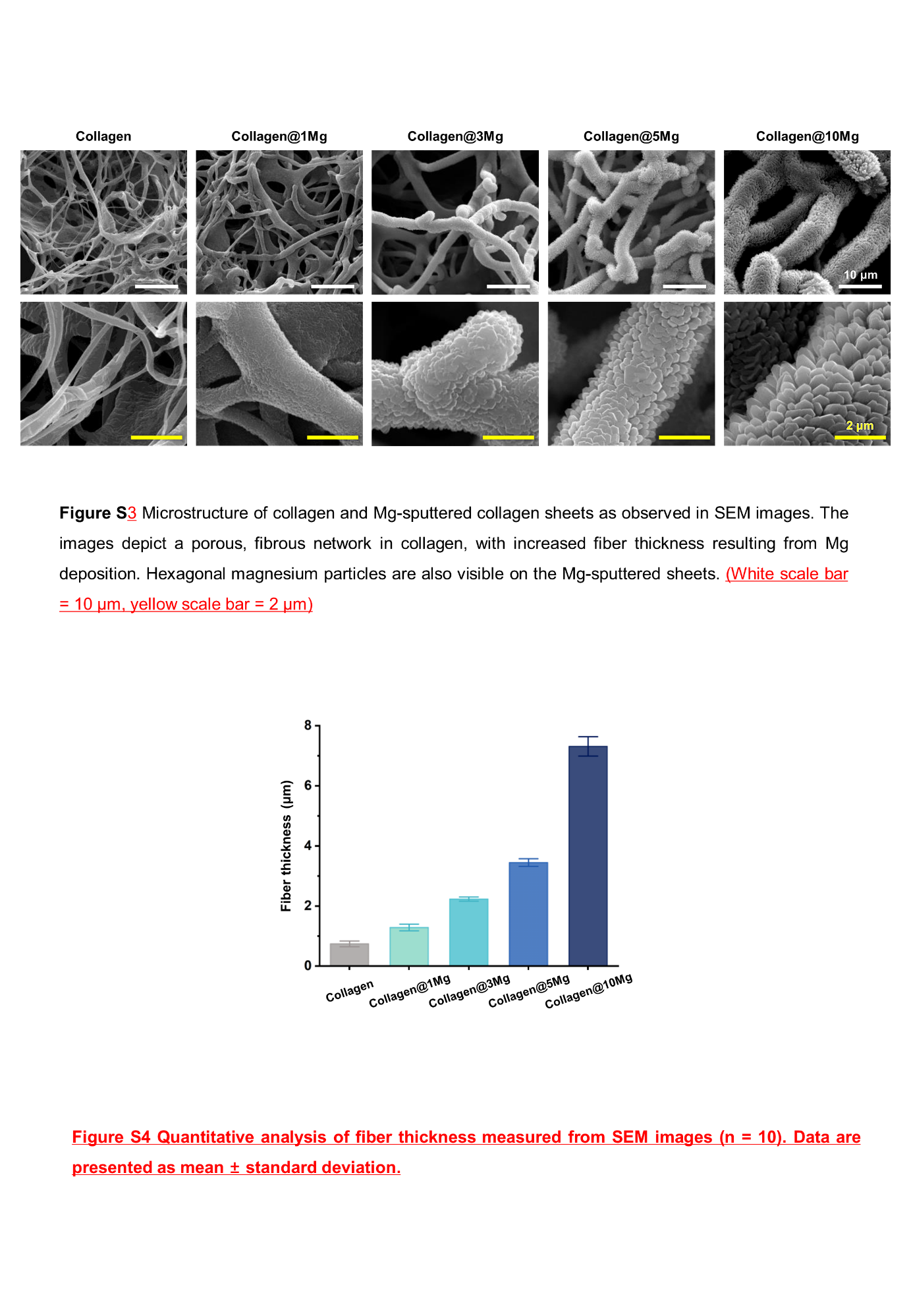


**Figure S3** Microstructure of collagen and Mg-sputtered collagen sheets observed in SEM images. The collagen sheet exhibits a porous, fibrous network, while the Mg-sputtered collagen sheet shows increased fiber thickness due to Mg deposition. Hexagonal magnesium particles are visible on the Mg-sputtered sheets. (White scale bar = 10 μm, yellow scale bar = 2 μm)


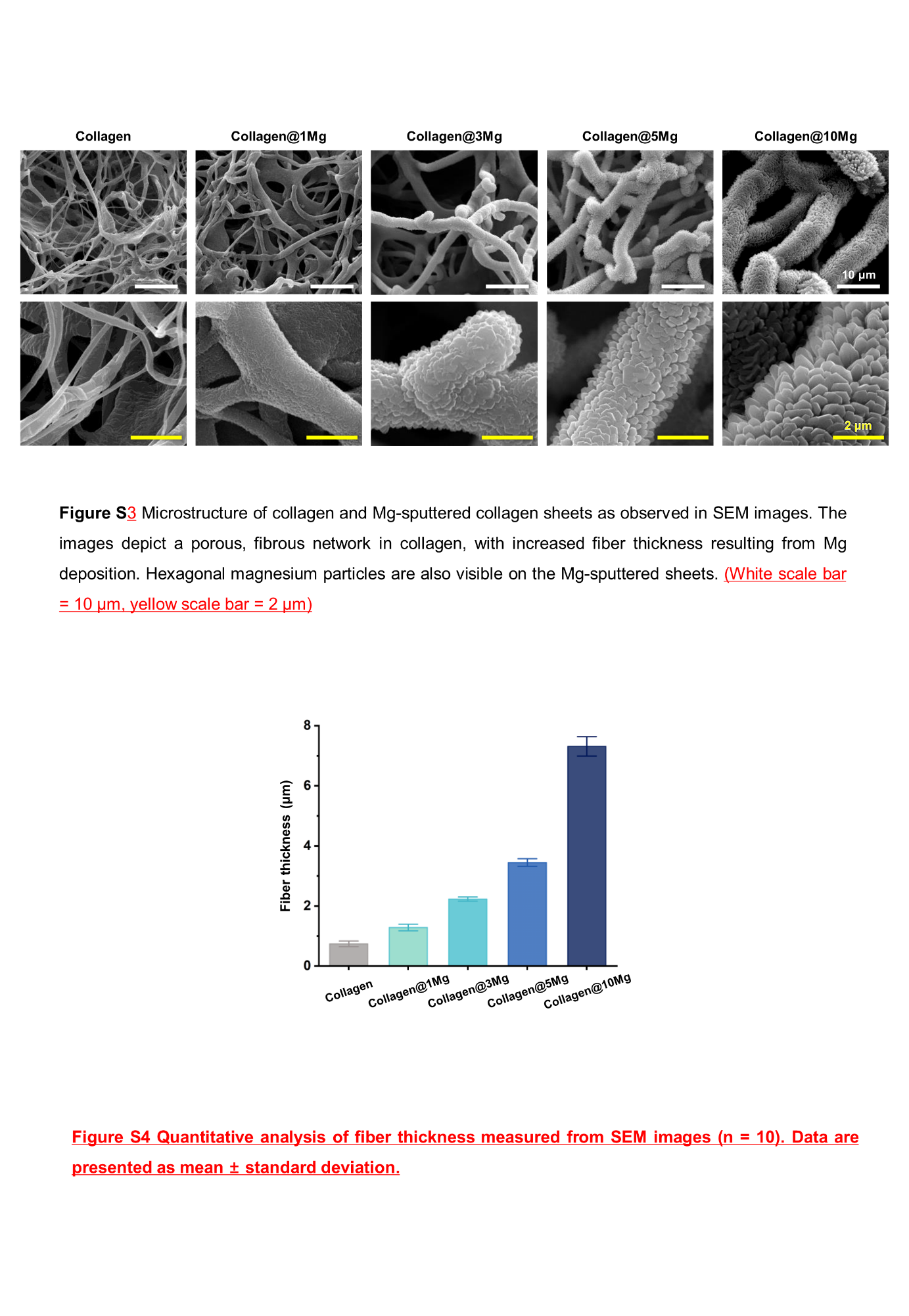


**Figure S4** Quantitative analysis of fiber thickness measured from SEM images (n = 10). Data are presented as mean ± standard deviation.


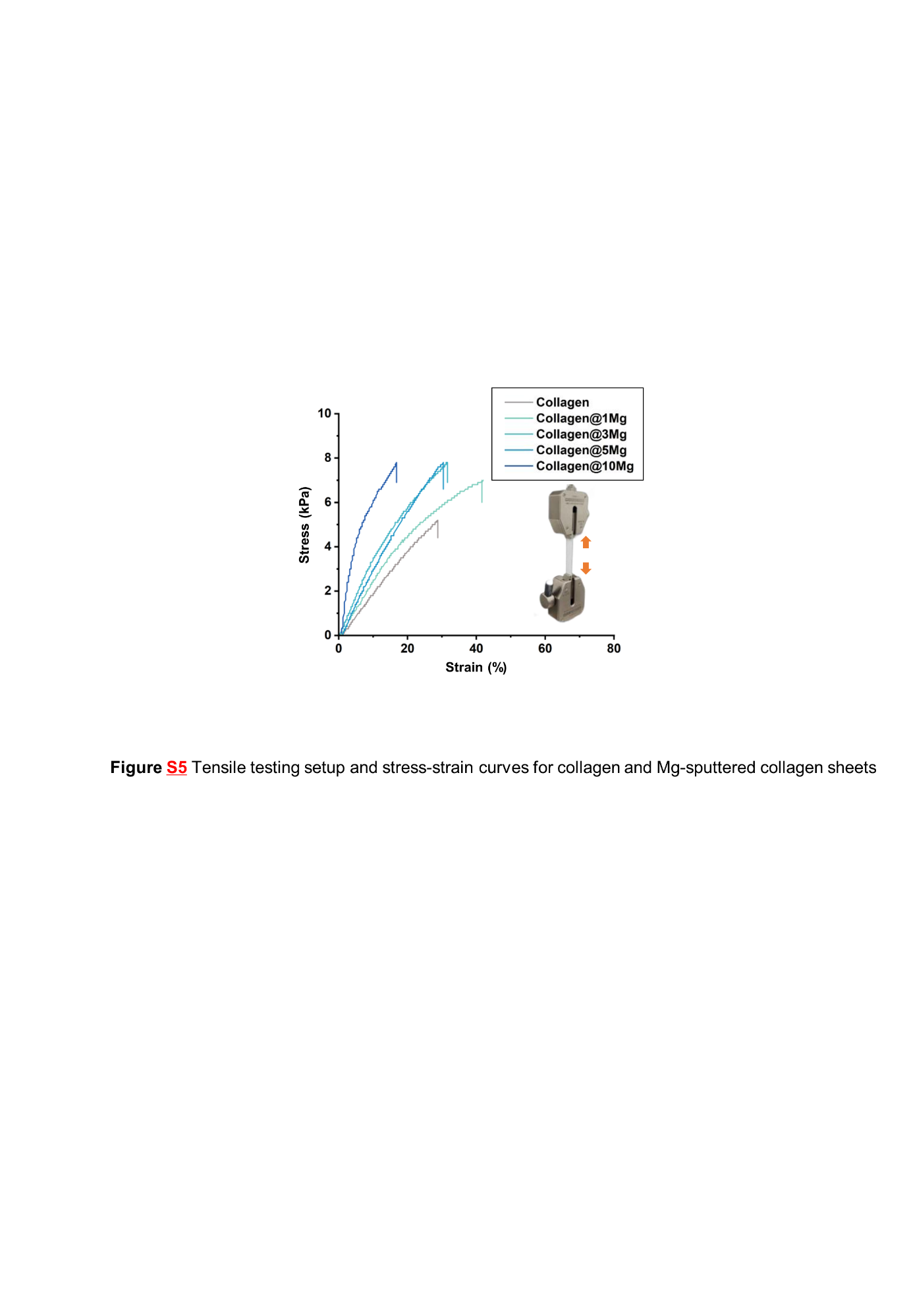


**Figure S5** Tensile testing setup and stress-strain curves for collagen and Mg-sputtered collagen sheets.

**
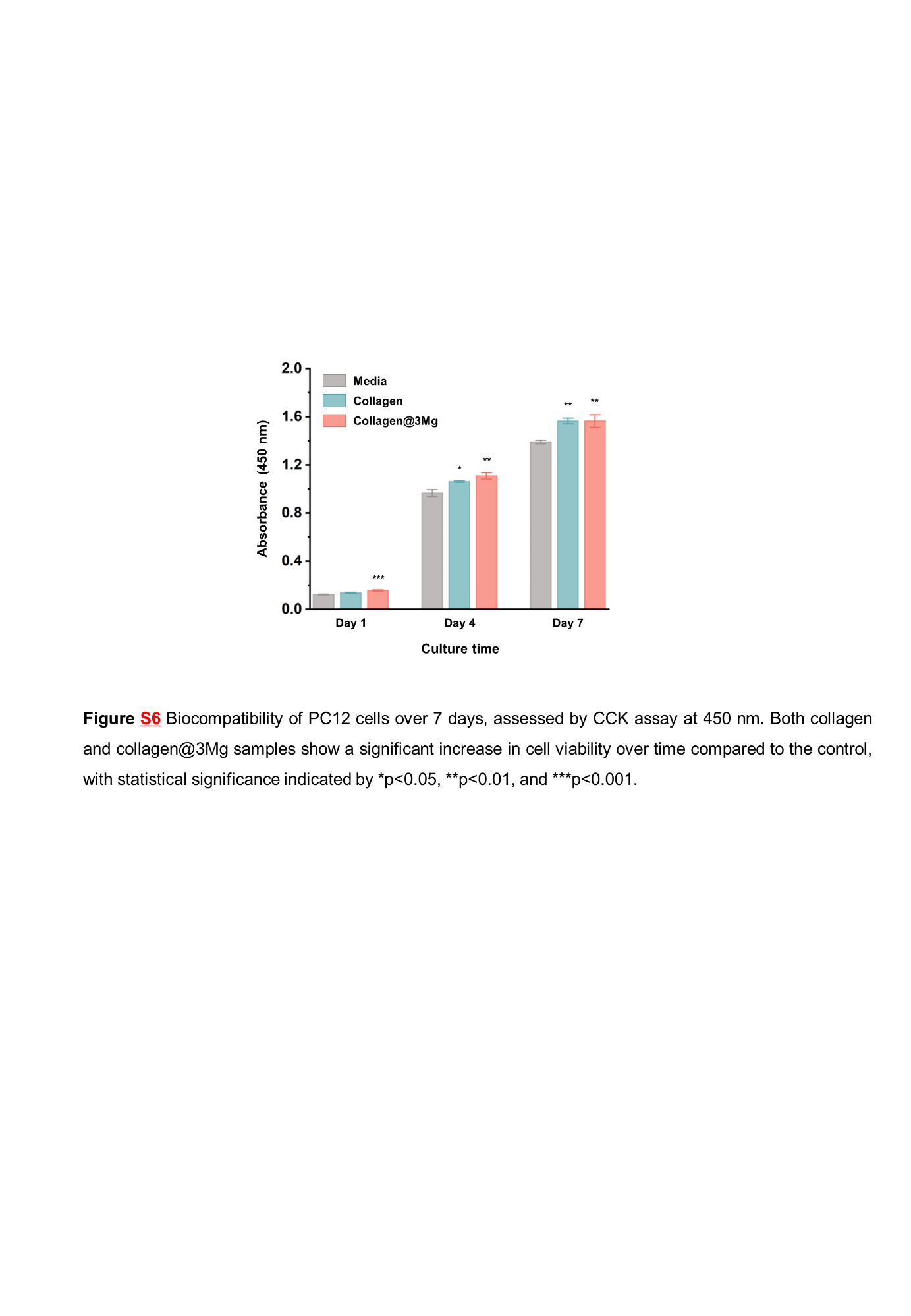
**

**Figure S6** Biocompatibility of PC12 cells over 7 days, assessed by the CCK-8 assay at 450 nm. Both collagen and collagen@3Mg samples show a significant increase in cell viability over time compared to the control. Data are presented as mean ± SEM, with *p<0.05, **<0.01, and ***p<0.001 indicating significance compared to the control, as determined by ANOVA followed Tukey’s test.


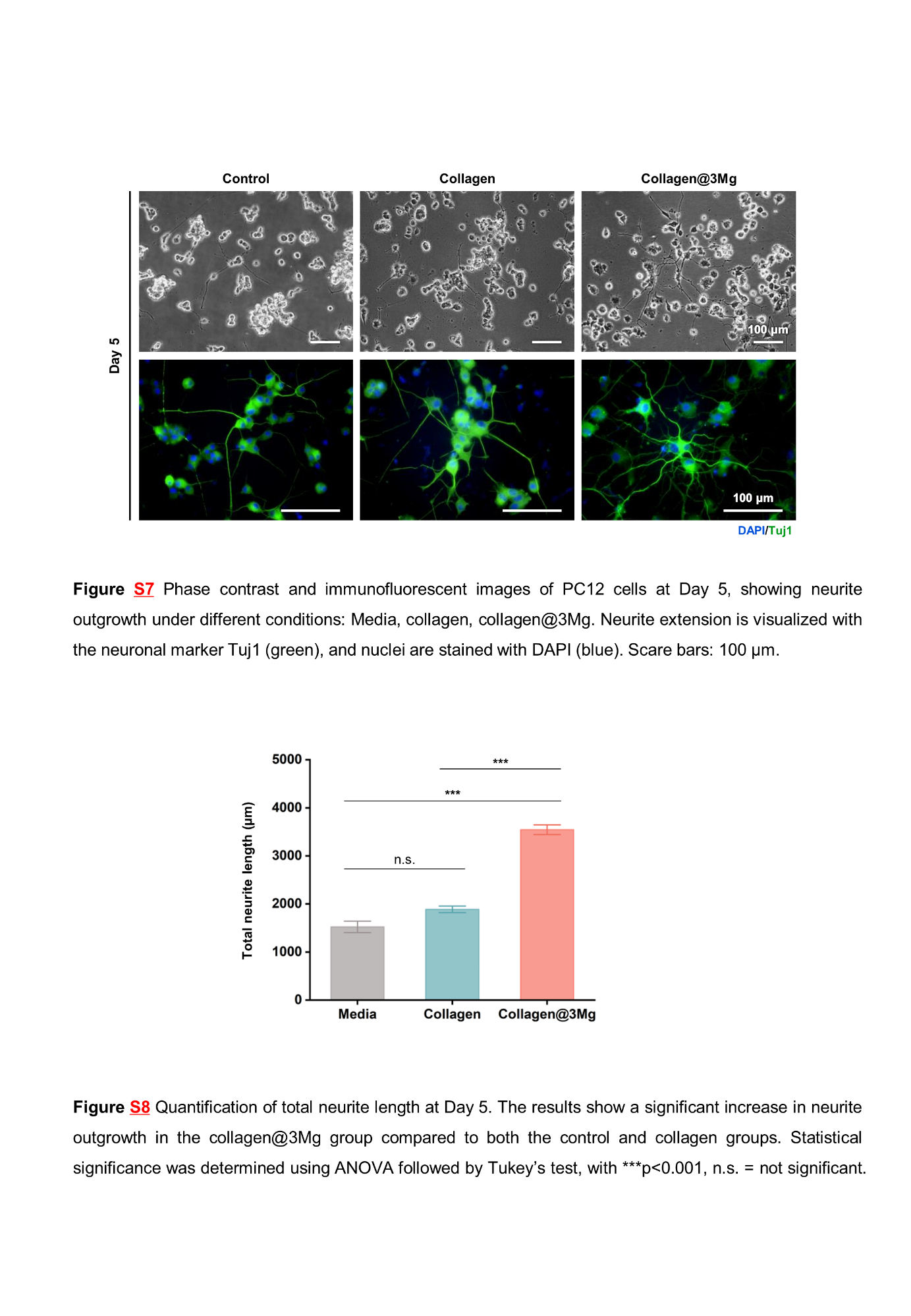


**Figure S7** Phase contrast and immunofluorescent images of PC12 cells at Day 5, illustrating neurite outgrowth under different conditions: Media, collagen, collagen@3Mg. Neurite extension is visualized using the neuronal marker Tuj1 (green), and nuclei are stained with DAPI (blue). Scale bars were white lines indicating 100 μm.


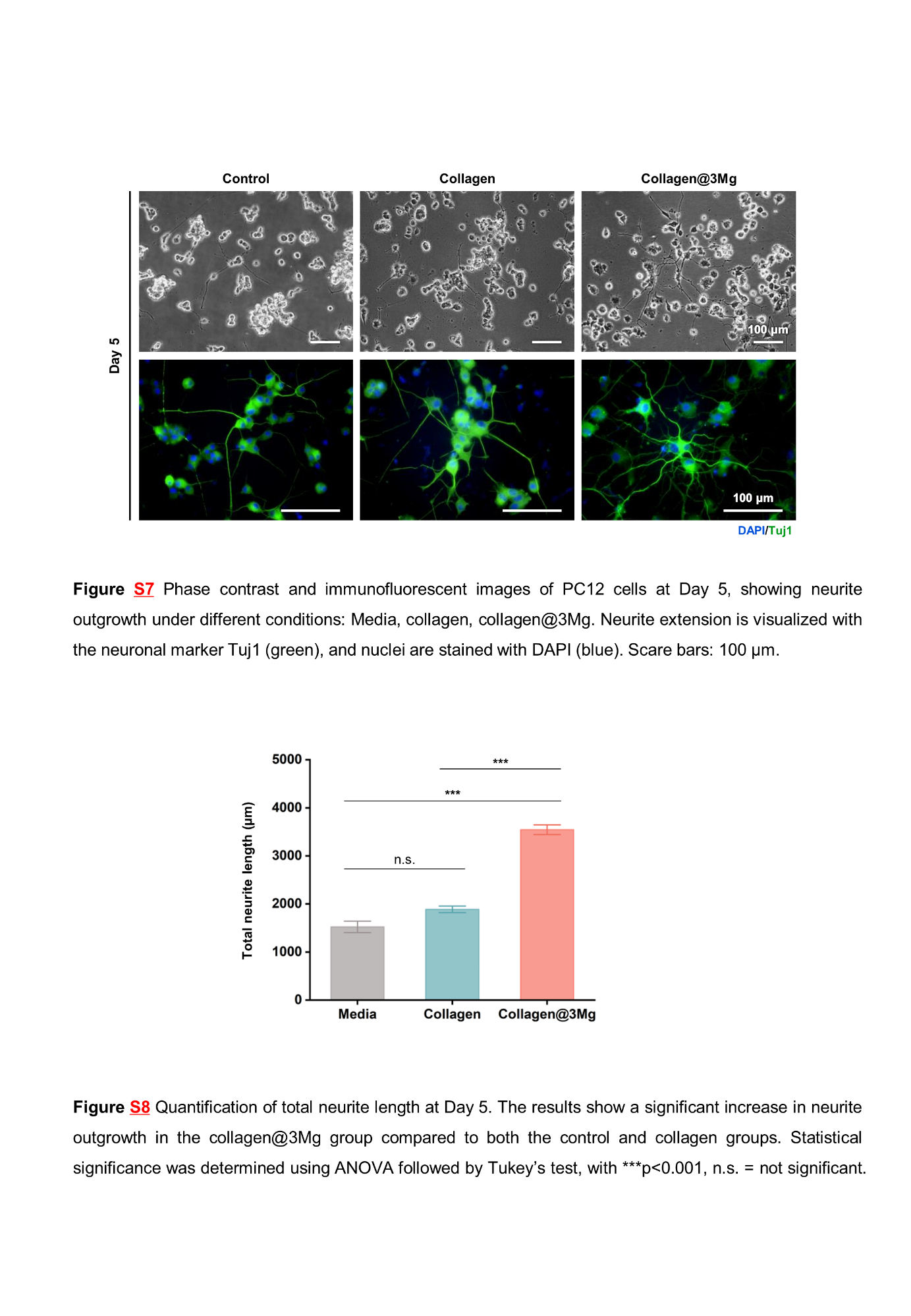


**Figure S8** Quantification of total neurite length at Day 5. The results show a significant increase in neurite outgrowth in the collagen@3Mg group compared to the control and collagen groups. Data are presented as mean ± SEM, with ***p<0.001 indicating significance compared to the control, as determined by ANOVA followed Tukey’s test, n.s. = not significant.


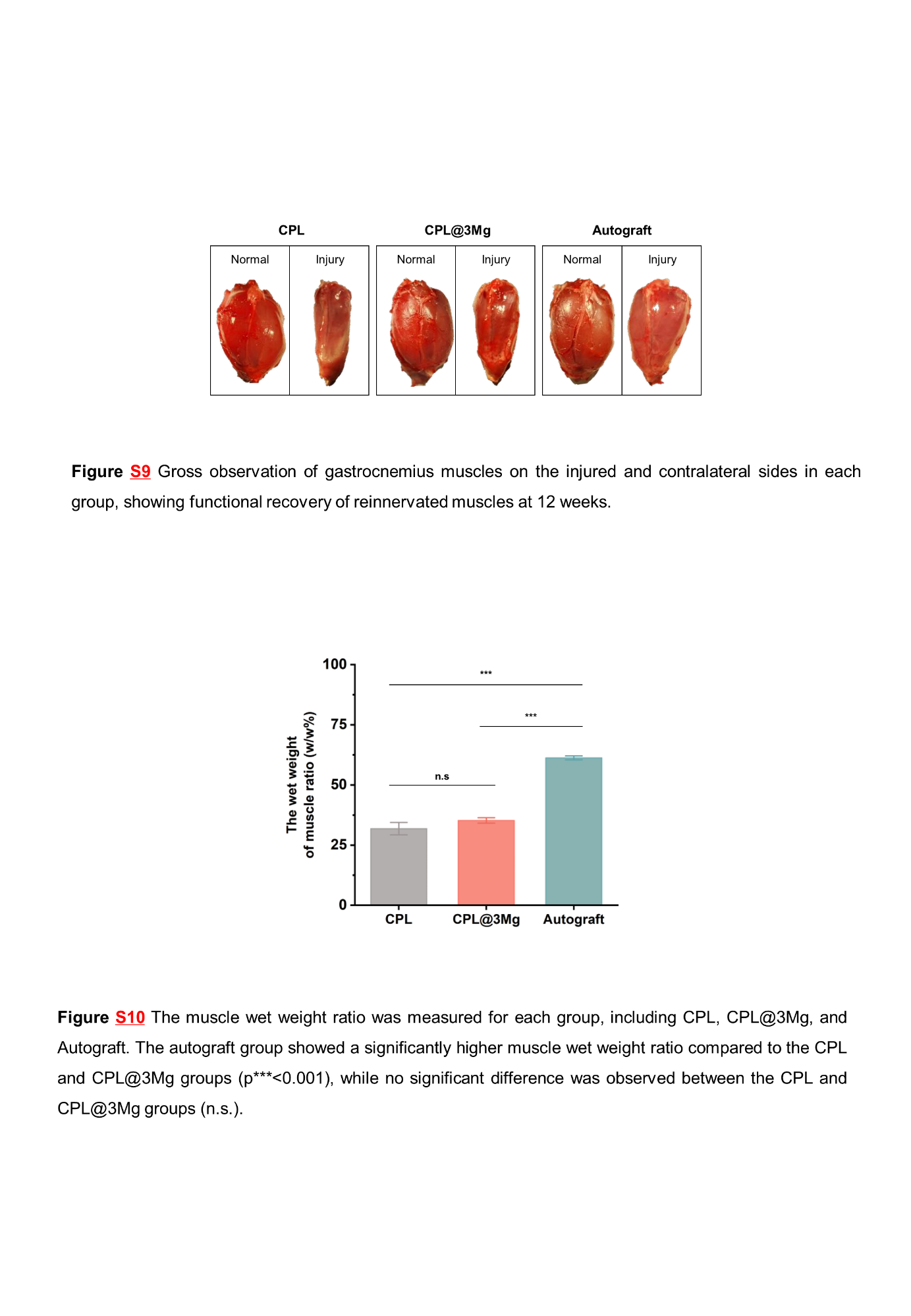


**Figure S9** Gross observation of gastrocnemius muscles on the injured and contralateral sides in each group, illustrating the functional recovery of reinnervated muscles at 12 weeks.


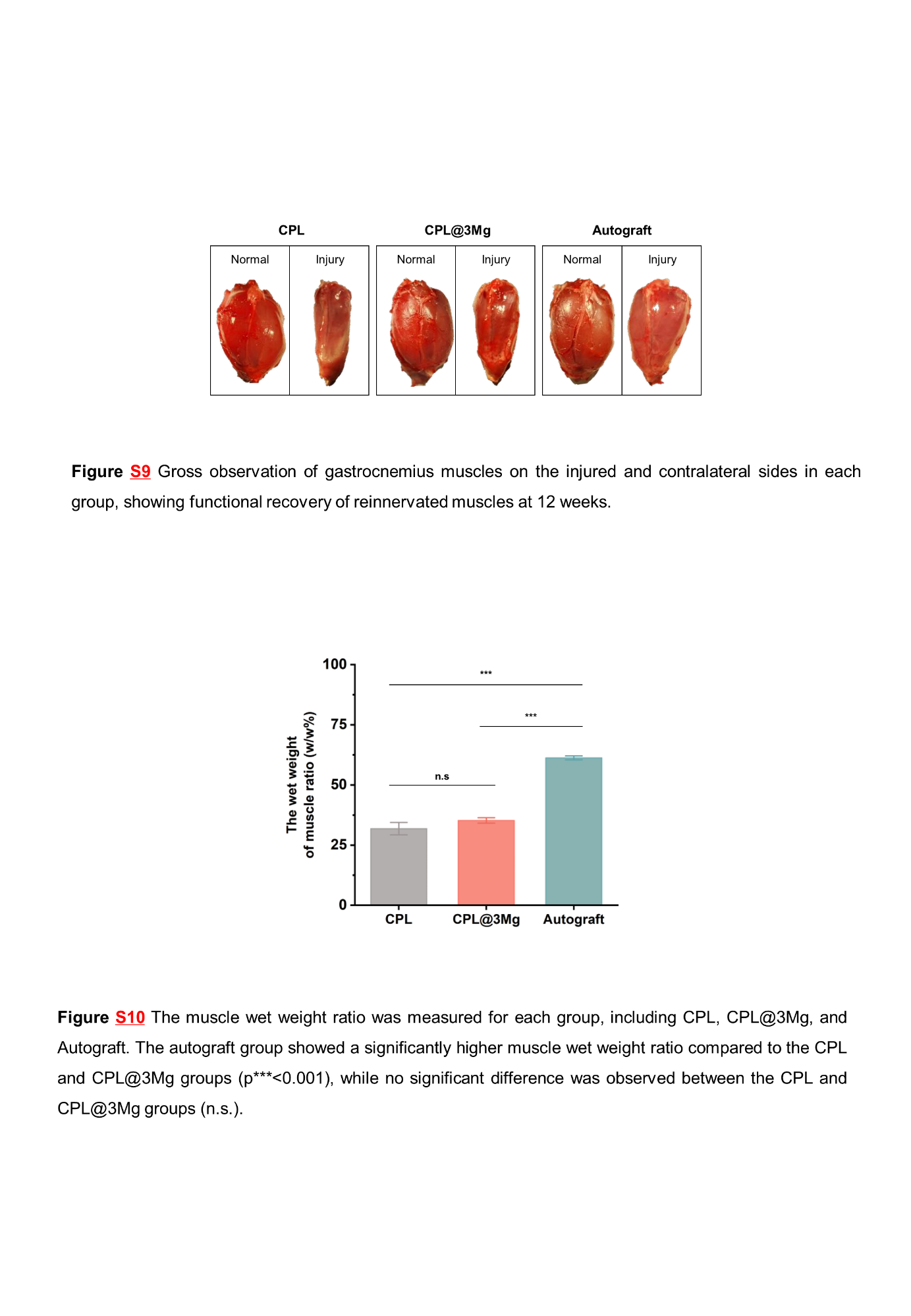


**Figure S10** The muscle wet weight ratio measured for each group: CPL, CPL@3Mg, and autograft. The autograft group showed a significantly higher muscle wet weight ratio compared to the CPL and CPL@3Mg groups, while no significant difference was observed between the CPL and CPL@3Mg groups. Data are presented as mean ± SEM, with ***p<0.001 indicating significance compared to the control, as determined by ANOVA followed Tukey’s test, n.s. = not significant.


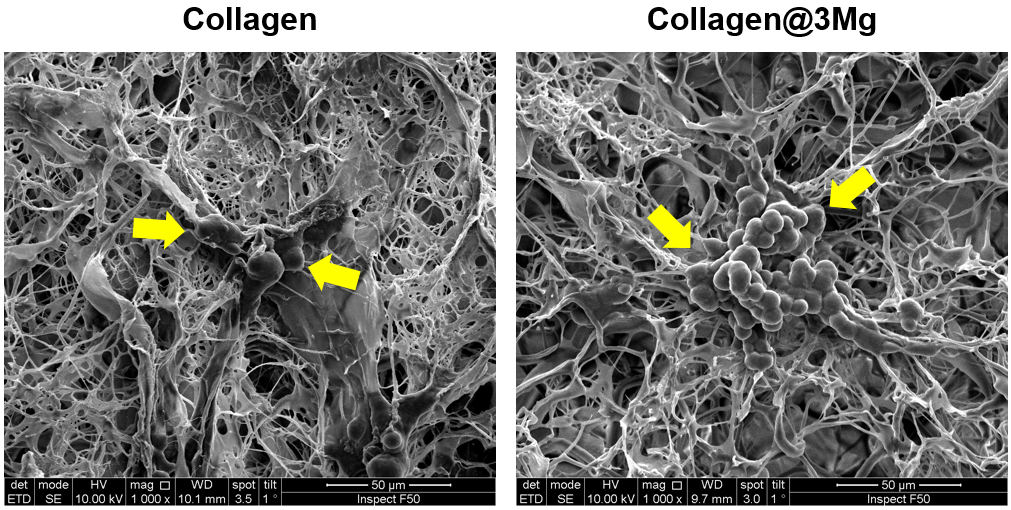


**Figure S11** Supplementary figure for the reviewer: SEM images of PC12 cells cultured on collagen (CPL) and collagen@3Mg (CPL@3Mg) at day 4. Yellow arrows indicate PC12 cells. This figure is provided for reviewer reference only and is not included in the manuscript.
